# Lipophilic activated ester prodrug approach for drug delivery to the intestinal lymphatic system

**DOI:** 10.1016/j.jconrel.2018.07.022

**Published:** 2018-09-28

**Authors:** Jong Bong Lee, Atheer Zgair, Jed Malec, Tae Hwan Kim, Min Gi Kim, Joseph Ali, Chaolong Qin, Wanshan Feng, Manting Chiang, Xizhe Gao, Gregory Voronin, Aimie E. Garces, Chun Long Lau, Ting-Hoi Chan, Amy Hume, Tecashanell M. McIntosh, Fadi Soukarieh, Mohammed Al-Hayali, Elena Cipolla, Hilary M. Collins, David M. Heery, Beom Soo Shin, Sun Dong Yoo, Leonid Kagan, Michael J. Stocks, Tracey D. Bradshaw, Peter M. Fischer, Pavel Gershkovich

**Affiliations:** a School of Pharmacy, University of Nottingham, Nottingham NG7 2RD, UK; b College of Pharmacy, University of Anbar, Anbar 31001, Iraq; c DMPK, Evotec, Milton Park, Abingdon, Oxfordshire OX14 4RZ, UK; d College of Pharmacy, Catholic University of Daegu, Gyeongsan 38430, Republic of Korea; e School of Pharmacy, Sungkyunkwan University, Suwon 16419, Republic of Korea; f Department of Pharmaceutics, Ernest Mario School of Pharmacy, Rutgers, The State University of New Jersey, Piscataway, NJ 08854, USA; g Comparative Medicine Resources, Rutgers, The State University of New Jersey, Piscataway, NJ 08854, USA; h School of Pharmacy, Universita di Roma Tor Vergata, Rome 00173, Italy

**Keywords:** Lymphatic transport, Prodrugs, Bexarotene, Retinoic acid, Chylomicron, Activated esters

## Abstract

The intestinal lymphatic system plays an important role in the pathophysiology of multiple diseases including lymphomas, cancer metastasis, autoimmune diseases, and human immunodeficiency virus (HIV) infection. It is thus an important compartment for delivery of drugs in order to treat diseases associated with the lymphatic system. Lipophilic prodrug approaches have been used in the past to take advantage of the intestinal lymphatic transport processes to deliver drugs to the intestinal lymphatics. Most of the approaches previously adopted were based on very bulky prodrug moieties such as those mimicking triglycerides (TG). We now report a study in which a lipophilic prodrug approach was used to efficiently deliver bexarotene (BEX) and retinoic acid (RA) to the intestinal lymphatic system using activated ester prodrugs. A range of carboxylic ester prodrugs of BEX were designed and synthesised and all of the esters showed improved association with chylomicrons, which indicated an improved potential for delivery to the intestinal lymphatic system. The conversion rate of the prodrugs to BEX was the main determinant in delivery of BEX to the intestinal lymphatics, and activated ester prodrugs were prepared to enhance the conversion rate. As a result, an 4-(hydroxymethyl)-1,3-dioxol-2-one ester prodrug of BEX was able to increase the exposure of the mesenteric lymph nodes (MLNs) to BEX 17-fold compared to when BEX itself was administered. The activated ester prodrug approach was also applied to another drug, RA, where the exposure of the MLNs was increased 2.4-fold through the application of a similar cyclic activated prodrug. Synergism between BEX and RA was also demonstrated *in vitro* by cell growth inhibition assays using lymphoma cell lines. In conclusion, the activated ester prodrug approach results in efficient delivery of drugs to the intestinal lymphatic system, which could benefit patients affected by a large number of pathological conditions.

## Graphical abstract

**Figure:**
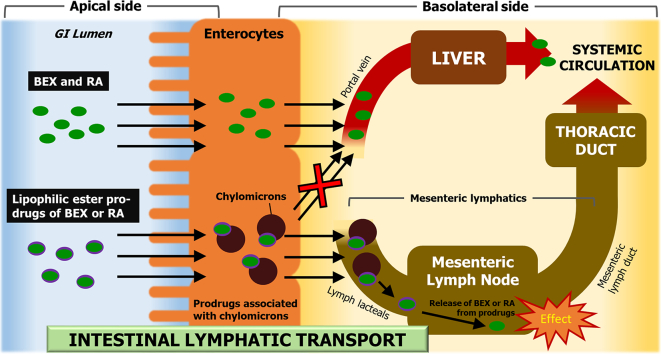

## 1 Introduction

The intestinal lymphatic system is an important organ of the immune system as it accommodates more than half of the body's lymphocytes [1, 2]. In addition, the intestinal lymphatic system plays an important role in the pathophysiology of multiple diseases including lymphomas, metastasis of some solid tumours, and human immunodeficiency virus (HIV) infection. Therefore, efficient delivery of drugs to the intestinal lymphatic system has potential to improve treatment of diseases such as autoimmune disorders, lymphatic system-associated cancers, HIV infections and cancer metastasis [[3], [4], [5], [6]]. However, only a very limited proportion of a drug can usually be distributed from the systemic circulation into the lymphatic system [4, 7]. As a result, in order to achieve sufficient concentrations of the drugs in the affected lymph nodes, the required levels in systemic circulation would be very high and associated with significant adverse side effects. Therefore, there is an unmet need for specific delivery of therapeutic agents to the intestinal lymphatics for treatment of patients affected by diseases associated with the intestinal lymphatic system.

**Fig. 1:**
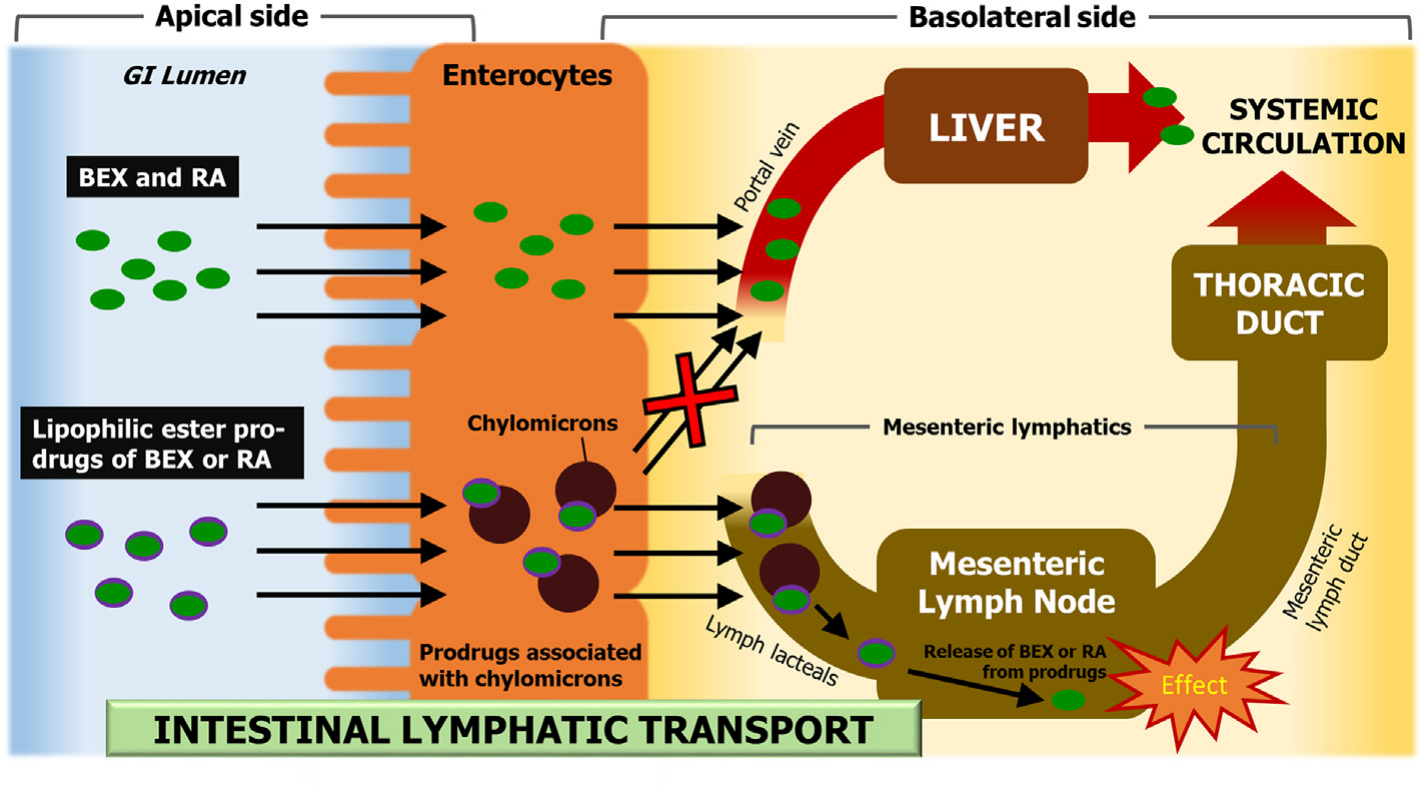
Schematic diagram of the intestinal lymphatic transport pathway. Highly lipophilic drugs and prodrugs with appropriate physicochemical properties are able to associate with the chylomicrons (CM) in the enterocyte. The drug-CM complex is too large to penetrate the blood capillaries and therefore is passed on to the intestinal lymphatic system before reaching the systemic circulation. GI, gastrointestinal; BEX, bexarotene; RA, retinoic acid.

Although most drugs absorbed from the gastrointestinal (GI) system are passed to the portal vein, lipophilic compounds may also gain access to systemic circulation through the intestinal lymphatics [8, 9] (Fig. 1). This distribution to the lymphatic system is determined mainly by the association of drugs with large lipoproteins, *i.e.* chylomicrons (CM), in the enterocytes. This is because drug molecules need to be associated with CM in order to utilise them as a carrier to the lymphatic system. In fact, a linear correlation between drug association with CM and lymphatic absorption is well established [10].

Intestinal lymphatic transport has been studied previously as an absorption pathway, with a primary focus on the fact that this absorption pathway can bypass the liver at the first pass following enteric drug administration, hence decreasing first-pass metabolism and thereby increasing the systemic bioavailability of drugs [[11], [12], [13], [14]]. However, an issue that has been overlooked in the past is that the lymphatic system has functional importance, and drugs that are transported by this route can reach very high concentrations in lymph fluid and lymph nodes, and can therefore exert their pharmacological activity within the lymphatic system itself [12]. It is known that if a drug has the necessary physicochemical properties, an appropriate lipid-based formulation can facilitate the transport of the drug *via* the intestinal lymphatic system following oral administration [5, 15]. On the other hand, lipophilic prodrug approaches have been employed in the past to take advantage of the intestinal lymphatic transport of drug molecules that otherwise would not have the necessary physicochemical properties required for association with CM. However, previously suggested approaches were mainly based on glyceride mimetic or very bulky prodrug moieties [8, 14, [16], [17], [18]]. It was believed that an alkyl ester prodrug approach would not be suitable for this purpose because of the instability of alkyl esters during the absorption phase [8].

We now report a study where a lipophilic prodrug approach was used to deliver bexarotene (BEX) and retinoic acid (RA) efficiently to the intestinal lymphatic system by a novel activated ester prodrug approach. Drugs with a wide range of indications and mechanisms of action could potentially benefit from delivery to the intestinal lymphatic system by the proposed approach. Here we focused on delivery of BEX and RA to potentially improve the treatment outcomes of non-Hodgkin's lymphoma (NHL) in patients with substantial mesenteric lymph node (MLN) involvement in the disease. Lymphoma is the most common cause of mesenteric lymphadenopathy [19]. In as many as 30–50% of NHL patients, especially those with diffuse large B-cell lymphoma (DLBCL), disease significantly affects MLNs [6]. This lymphadenopathy is persistent even in the disease remission stage, rendering patients susceptible to relapse [19]. In this study, 25 prodrugs of BEX were synthesised and assessed. The activated ester prodrug was shown as the most promising approach for efficient delivery to the intestinal lymphatic system. This successful approach was further applied to RA. In addition, our results show that the two drugs exert synergy in treatment of DLBCL *in vitro* at concentrations which are realistically achievable in the lymph nodes, when the suggested delivery approach is applied *in vivo*.

## 2 Materials and methods

### 2.1 Materials

BEX was purchased from LC Laboratories (Woburn, MA, USA). RA (all-*trans*), Intralipid®, esterase from porcine liver, sodium taurocholate (NaTc), NaCl, NaF and lecithin were obtained from Sigma (Gillingham, UK). Rat plasma was purchased from Sera Laboratories (West Sussex, UK). Thiazolyl blue tetrazolium bromide (MTT), sesame oil, polyethylene glycol 400 (PEG400) and all solvents (HPLC grade or higher) were purchased from Fisher Scientific (Loughborough, UK).

### 2.2 Chemical synthesis

#### 2.2.1 General synthetic scheme for esterification

Ester prodrugs of BEX and RA were synthesised by adding 2.87 mmol of the desired alcohol to 0.287 mmol of BEX or RA. 1-Ethyl-3-(3-dimethylaminopropyl) carbodiimide (0.431 mmol) and 4-dimethylaminopyridine (0.057 mmol) were added, and dichloromethane (15 mL) was used as the solvent. The mixture was stirred magnetically overnight at room temperature. Purification was performed by flash chromatography using a 200–400 mesh silica gel-packed glass columns and hexane–ethyl acetate 50:2 (*v*/v) as the mobile phase. Specific details for compounds that were obtained by different synthetic schemes are described in Supplementary Material 1.

#### 2.2.2 Characterisation of synthetic prodrugs

^1^H NMR and ^13^C NMR spectra were obtained using a Bruker 400 Ultrashield instrument at 400 and 100 MHz, respectively. Bruker TOPSPIN 2.1 software was used to analyse the spectra. Chemical shifts are reported as parts per million (ppm) relative to tetramethylsilane (internal standard) to the nearest 0.01 ppm and coupling constants (*J*) to the nearest 0.1 Hz.

### 2.3 CM and artificial lipid particle association assay

#### 2.3.1 Preparation of artificial lipid particle emulsion for *in vitro* association studies

To prepare the artificial lipid particle emulsion, Intralipid® 20% (Sigma, Gillingham, UK) was diluted using phosphate buffered saline (PBS) to yield 100 mg/dL of triglyceride (TG) concentration [15, 20]. A TG enzymatic kit (Sigma Aldrich, Dorset, UK) was used to monitor the TG concentration by following manufacturer's instructions.

#### 2.3.2 Preparation of human plasma-derived CM emulsion for *ex vivo* association studies

The protocol for preparation of human plasma-derived CM emulsion was approved by the Faculty of Medicine and Health Sciences Research Ethics Committee, Queens Medical Centre, Nottingham University Hospitals (BT12102015 CBS SoP). Healthy male volunteers of 19–40 years old and with body mass index of 18.5–25.0 were enrolled in the study after providing informed consent. The participants were excluded if any medication was used within one week prior to enrolment. On the morning of the study, participants received a high-fat meal (equivalent to a full English breakfast). Between 3 and 4 h following breakfast, 30 mL of blood was collected using heparinised tubes (Vacutainer® Blood Collection Tubes) and centrifuged (800 *g*, 10 min, 15 °C) to obtain plasma.

CM separation was performed as previously reported with minor modifications [10, 15]. Briefly, KBr (0.57 g) was mixed with 4 mL of plasma to yield density of 1.1 g/mL. PBS with densities of 1.006, 1.019, and 1.063 g/mL were prepared by adding appropriate amounts of KBr. These PBS of different densities were layered on top of the plasma to build density gradient layers in polyallomer ultracentrifuge tubes. The samples were then centrifuged at 268,350 *g* for 35 min at 15 °C using an ultracentrifuge (SORVALL® TH-641 Rotor, Thermo Fisher Scientific, UK). Following ultracentrifugation, approximately 1 mL of the upper layer containing CM was collected. TG concentration of CM emulsion was assessed using the TG enzymatic kit (Sigma Aldrich, Dorset, UK). TG concentration of the emulsion was adjusted to 100 mg/dL by diluting with PBS of 1.006 g/mL density. The CM emulsion was stored at 4 °C until the association assay (˂24 h).

#### 2.3.3 Association assay

The association of the compounds with artificial lipid particle emulsion and human CM was assessed using previously reported methodology with minor modifications [10]. Briefly, 1 mL of artificial lipid particle or CM emulsions (TG concentration 100 mg/dL) was used for the association assay. The emulsion was spiked with stock solutions of tested compounds (in acetonitrile) to give a final concentration of 1.755 μM. The reaction mixtures were incubated at 37 °C for 1 h with magnetic stirring at 300 rpm. Following the incubation, density gradient ultracentrifugation was applied as described above to isolate the artificial lipid particles or CM associated with the tested compounds from the rest of the experimental medium. All experiments were conducted in quintuplicates or above.

### 2.4 Prodrug conversion assay

The prodrugs were tested for their stability and conversion into their corresponding active drug using fasted state simulated intestinal fluid (FaSSIF) with added esterase enzyme activity (20 IU/mL), rat plasma, and postprandial rat lymph [21]. The FaSSIF was prepared as previously described in the literature [22]. The medium was pre-heated to 37 °C prior to the assay for 5 min. The experiment was initiated by spiking stock solutions of prodrugs to give a final concentration of 10 μM of the prodrugs in the medium. When the stability of the artificial emulsion-associated prodrugs was tested in plasma, the association of compounds with artificial emulsion was performed prior to stability experiments and the concentrations of the compounds in the associated emulsion were measured. The emulsion was then spiked to give a concentration of 10 μM of the prodrugs in plasma. The reaction mixtures were incubated at 37 °C and shaken at 200 rpm on a temperature-controlled orbital shaker (Thermo Scientific MaxQ4000, Waltham, MA, USA) for 4 h. Samples were withdrawn at pre-determined time points, and the reaction was terminated by addition of acetonitrile. The samples were then subjected to sample preparation and analysis as described below. The natural log percentage of the prodrug concentration was plotted against incubation time, and the slope (*k*) was obtained from the plot. Half-life of active drug release (*t*_*1/2*_) was calculated by the following equation: *t*_*1/2*_ = −0.693/*k*. All experiments were conducted in triplicate.

### 2.5 Animal experiments

#### 2.5.1 Animals

The protocols for pharmacokinetic and biodistribution experiments in this study were reviewed and approved by the University of Nottingham Ethical Review Committee in accordance with the Animals [Scientific Procedures] Act 1986. Male Sprague Dawley rats (Charles River Laboratories, UK) weighing 350–380 g were used. The animals were housed in the Bio Support Unit, University of Nottingham. Continuous lymph collection was performed according to a protocol approved by the Institutional Animal Care and Use Committee at Rutgers, The State University of New Jersey. Male Sprague Dawley rats (Envigo, Inc., NJ, USA) weighing 300–350 g were used for the continuous lymph collection. Animals were allowed free access to food and water and were acclimatised for at least 6 days prior to any procedures at both institutions in a controlled-temperature environment with 12 h light/dark cycles.

#### 2.5.2 Pharmacokinetic study

Jugular vein cannulation surgery procedures were conducted under general anaesthesia for blood sampling. The animals were allowed two days to recover following surgery and were fasted up to 12 h prior to experiments with free access to drinking water. Formulations were prepared at concentrations equivalent to 2.5 mg/mL and 10 mg/mL of active drug for intravenous and oral administration, respectively. Solutions of the tested compounds in PEG400 were used as the dosing vehicle for intravenous administration. For oral administration, all prodrugs were administered as a solution in sesame oil to stimulate intestinal lymphatic transport [15]. For oral administration of BEX and RA, oral gavage of sesame oil was administered prior to administration of BEX or RA solution in PEG400 due to low solubility of the two compounds in TG. In the lipid-free group BEX was administered orally formulated as a solution in PEG400. The formulations were administered by an oral gavage for oral administration or *via* the jugular vein cannula followed by 0.3 mL heparinised saline (50 IU/mL) for intravenous injection. Blood samples of 250 μL volume were collected from the cannula at pre-determined time points following administration. NaF (10 mg/mL) was used as an anticoagulant and inhibitor of esterase activity. Blood samples were centrifuged at 3000 *g* for 10 min to obtain plasma.

#### 2.5.3 Biodistribution study

Formulations were prepared as described in the above pharmacokinetic study and were delivered by an oral gavage. Animals were euthanised at pre-determined time points. Blood was collected from the posterior vena cava and lymph samples were taken from the mesenteric lymph duct. MLNs were dissected and collected as previously described [5, 23]. The MLNs were isolated from surrounding tissue with care not to compromise the integrity of the lymph nodes. The MLNs were homogenised (POLYTRON® PT 10–35 GT, Kinematica AG, Luzern, Switzerland) with 10 mg/mL NaF in water (1,2, *w*/*v*) on an ice bath prior to sample preparation for HPLC analysis.

#### 2.5.4 Postprandial continuous lymph collection

Surgical and experimental procedures for lymph collection were performed as previously described with minor modifications [24]. Peanut oil (2 mL) was orally administered to the animals 1–2 h prior to surgery. The thoracic duct was cannulated and the cannula was exteriorised under the skin to the back of the neck under general anaesthesia. The rats were allowed to recover for 24 h before collection and gel-based food and water were provided during recovery and lymph collection. Postprandial lymph was collected continuously through the extension tubing into a tube kept on ice.

### 2.6 TG solubility measurement

Compounds were dissolved in fresh aliquots of sesame oil in excessive amounts in glass vials (in triplicate) and stirred using a magnetic stirring bar at 37 °C for 72 h. The mixture was then filtered by centrifugation in a Costar Spin-X Centrifuge Tube (Fisher Scientific, Loughborough, UK) at 2400 *g* for 5 min. The filtrates were analysed for concentration of the tested compounds by means of HPLC.

### 2.7 Analytical methods for determination of concentrations of BEX, RA and their prodrugs in biological samples

#### 2.7.1 Sample preparation

A combination of protein precipitation and liquid-liquid extraction was applied to samples prior to HPLC injection. To 100 μL sample, 300 μL of acetonitrile was added for protein precipitation and then 300 μL of pH modifier was added when necessary (Supplementary Material Table S2). Liquid-liquid extraction was achieved with hexane (3 mL) by vortex-mixing for 10 min and centrifugation at 1160 *g* for 5 min. The organic layer was separated and evaporated under N_2_ gas. Reconstitution solvent (100 μL) was used as detailed in Supplementary Material Table S2 and 40 μL was injected into the HPLC-UV system. The stability of the prodrugs was preliminarily tested in plasma supplemented with 10 mg/mL NaF for 3 h at room temperature and 24 h at −20 °C, demonstrating that they were stable at these conditions (>90% stability). Therefore, storage and preparation of all samples were performed within the time frame and conditions of confirmed stability.

#### 2.7.2 Analytical conditions

The HPLC-UV system consisting of a separation module (Waters Alliance 2695) equipped with a UV detector (Waters 996) was used for analysis. Samples in the autosampler were maintained at 4 °C and the column at 40 °C. The stationary and mobile phases were used as specified in Supplementary Material Table S2. BEX and its prodrugs were detected at 259 nm and RA and its prodrugs at 357 nm.

### 2.8 Cell growth inhibition assay

DLBCL cell lines of DOHH2 and VAL were obtained from the Gene Regulation and RNA Biology group in University of Nottingham. Cell growth inhibition was assessed by MTT assay with a minor modification from previously reported methods [[25], [26], [27]]. Cells were seeded at a density of 2000 cells/well and allowed to adapt overnight. Test compounds were serial-diluted with dimethyl sulfoxide (DMSO) and spiked on the next day to yield 0.1% (*v*/v) DMSO. Control wells were treated with 0.1% DMSO vehicle alone. At the time of drug addition, separately prepared wells were processed for T_zero_ readings as previously described [27]. The treated cells were incubated further for 72 h at 37 °C and processed for cell growth assessments.

### 2.9 Statistical analyses

Assessment of statistical significance of differences between two groups was conducted by means of two-tailed unpaired *t*-test. Statistical significance among three or more groups was assessed by means of a one-way ANOVA followed by Tukey's multiple comparisons test. Statistical significance was declared when a *p*-value was lower than 0.05. The analyses were performed on GraphPad Prism version 7.01 (GraphPad Software, Inc., La Jolla, CA, USA). The concentrations that inhibited cell growth by 50% (*GI*_*50*_) were also calculated using GraphPad Prism and isobole plots were presented following a previously reported method to assess synergy between BEX and RA [28]. Pharmacokinetic parameters were calculated by non-compartmental analysis using Phoenix WinNonlin 6.3 software (Pharsight, Mountain View, CA, USA).

## 3 Results

### 3.1 Preparation of prodrugs of BEX

**Fig. 2:**
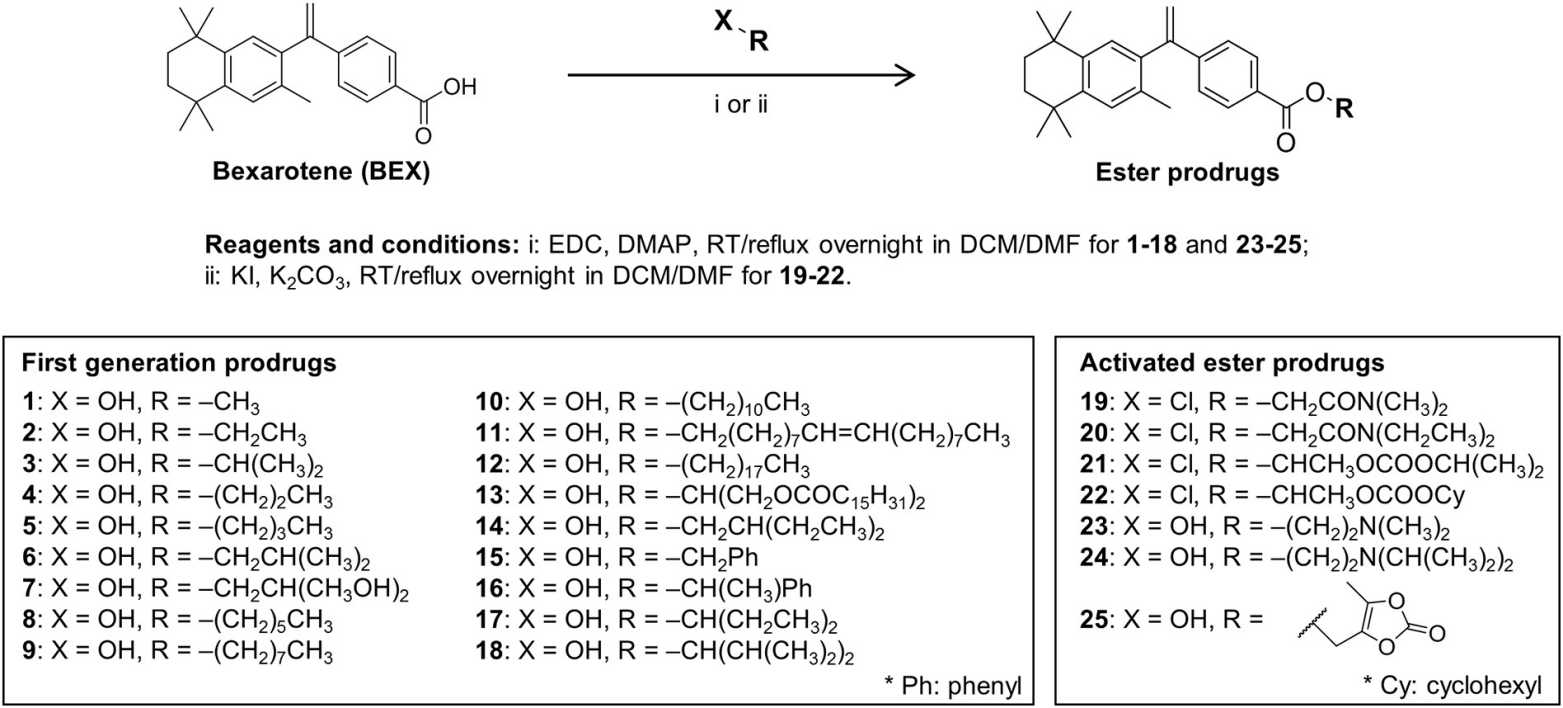
Chemical structures of bexarotene (BEX) and its prodrugs.

A total of 25 prodrugs of BEX were designed *in silico* to possess the necessary physicochemical properties needed for association with CM and therefore high potential for the intestinal lymphatic transport following oral administration [20]. All prodrugs were then successfully synthesised and purified (Fig. 2; compound characterisation in Supplementary Material 1). Prodrugs 1–18 were the first generation of candidate compounds which were originally synthesised and tested. The activated ester prodrugs 19–25 were then developed to introduce improvement to the first generation candidate compounds.

### 3.2 Association of BEX and its prodrugs with lipoproteins

**Fig. 3:**
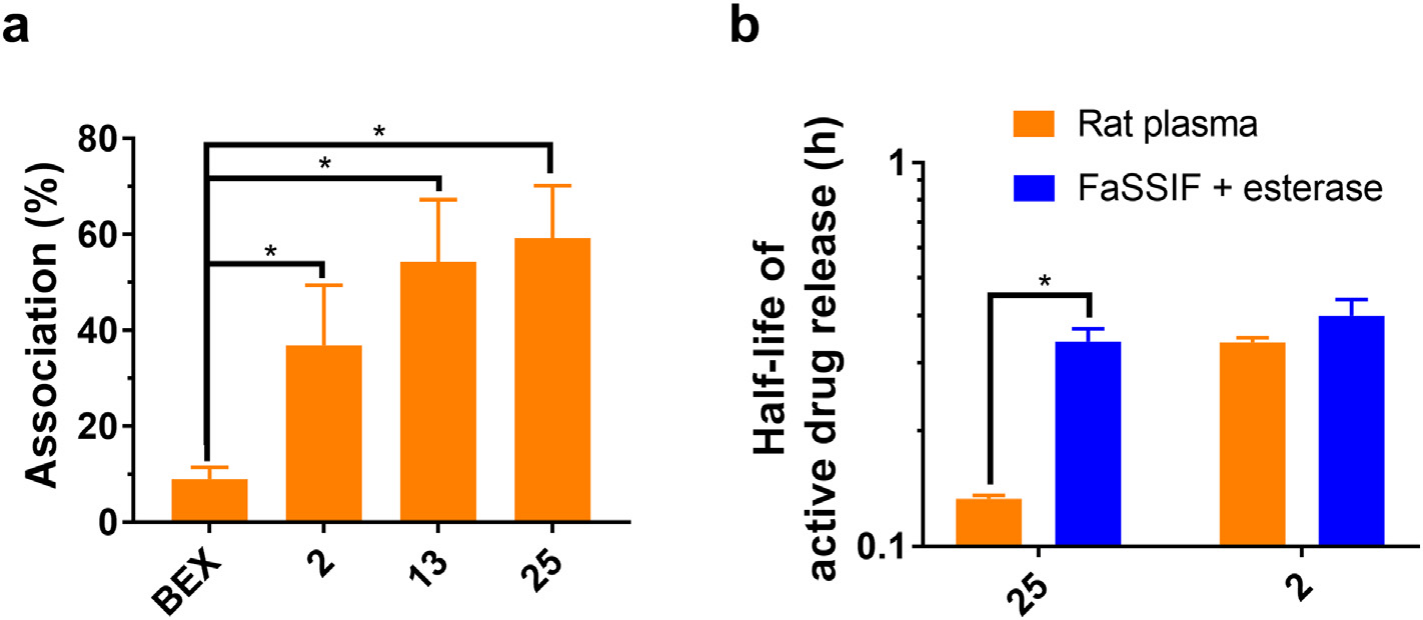
**Screening and selection of prodrug candidates for subsequent *in vivo* studies. (a)** Association of bexarotene (BEX) and its prodrugs selected for *in vivo* studies with human chylomicrons (CM) mean ± SD, *n* = 5). **(b)** Half-lives of active drug release from prodrugs of bexarotene (BEX) selected for *in vivo* studies in rat plasma and fasted state simulated intestinal fluid (FaSSIF) with added esterase activity (20 IU/mL) (mean ± SD, *n* = 3). BEX was not released from prodrug **13**. Full screening of all tested prodrugs including association with natural CM, artificial CM-like emulsion and stability in simulated intestinal fluid and plasma is shown in Supplementary Material (Fig. S1 and S3). *, *p* < .05.

The association of drugs with CM in the enterocytes is the key process that determines whether they can be delivered *via* the intestinal lymphatic transport pathway. Therefore the association was tested *in vitro* using artificial CM-like emulsion prepared from Intralipid®, as well as *ex vivo* using plasma-derived human CM. As shown in Fig. 3a and Supplementary Material Fig. S1, all prodrugs of BEX had higher association with CM than BEX (*p* < .05), indicating improved potential for delivery to the intestinal lymphatic system. The difference in affinity of compounds for the artificial emulsion *versus* natural human CM was not statistically significant for most prodrugs, except for prodrugs **20** and **24** (Supplementary Material Fig. S1). The correlation between the *in vitro* and *ex vivo* assessments demonstrated an *R*^*2*^ value of 0.694 (Supplementary Material Fig. S2). Intralipid® is a lipid emulsion mostly consisting of TG, and therefore the similarity in association suggests that the affinity to TG is the most important factor in the association of compounds with CM. However, prodrugs **20** and **24** showed significant differences (Supplementary Material Fig. S1), which indicates that surface apolipoproteins may play a role in association with CM at least for some molecules [20].

### 3.3 Conversion of BEX prodrugs to BEX

The active moiety should be released from the prodrug within the lymphatic system in order to achieve pharmacological effect on targets in the lymphatic system. In this work the candidate prodrugs were tested for their stability and *in vitro* conversion to the active drug (BEX) in rat plasma, which represented a reasonable surrogate for the enzymatic release of BEX upon reaching the intestinal lymphatic system (Fig. 3b and Supplementary Material Fig. S3). Since the main interest of this study is delivery of active drug to the intestinal lymphatic system, it would be, in theory, ideal to assess release of the active moiety directly in intestinal lymph fluid for all prodrug candidates. However, since we had above 25 prodrug candidates in this work, there are practical limitations of lymph volume that can be collected, which would result in excessive number of animals required, as well as technical and ethical issues associated with lymphatic cannulation surgery. Although slightly lower protein binding has been observed in lymph compared to plasma for some drugs, it is known that the composition and levels of enzymes in the lymph fluid is similar to those in blood [29, 30]. Therefore, rat plasma was chosen as a surrogate model to initially screen candidate prodrugs for the enzymatic release of the active moiety.

The *in vitro* conversion of the prodrugs was also tested in FaSSIF with added esterase enzyme activity (Fig. 3b and Supplementary Material Fig. S3), which is representative for release of the active drug from the prodrugs in the GI environment prior to absorption. All prodrugs had an ester bond which can be cleaved by esterases to release BEX. In both rat plasma and FaSSIF with esterase, the degradation of prodrugs corresponded to generation of BEX and no other product peaks were apparent in the HPLC-UV chromatograms at a wavelength of 259 nm upon bioanalysis, indicating direct biotransformation of the prodrugs to BEX. Therefore, the half-lives shown in Fig. 3b and Supplementary Material Fig. S3 represent the stability and conversion rate of the prodrugs to BEX. Prodrugs 10–13 did not degrade and did not release BEX in either medium within the time frame tested, therefore they were not included in the graph. The prodrugs 9 and 18 are the bulkiest compounds included in Supplementary Material Fig. S3, and probably for this reason their conversion in FaSSIF with esterase was too slow to be detected during the experiment, and hence half-life could not be calculated.

### 3.4 Selection of first generation prodrug candidates for *in vivo* studies

The criteria for selection of prodrug for further development in this work was high association with CM, good stability in the GI tract prior to absorption and immediate release of the active drug once it reaches the intestinal lymphatics. That being said, it was noted that drugs can still be orally bioavailable even with a relatively short half-life in GI fluids [31]. In addition, lipid-based formulations can hinder enzymatic reactions in the GI tract [32]. Therefore, two main properties, including high CM association and short half-life of active drug release in rat plasma were considered as the main attribute in prodrug selection. Among the first generation prodrugs (prodrugs **1**–**18**, before prodrugs **19**–**25** were designed), prodrug **2** (ethyl ester prodrug of BEX) was selected for further *in vivo* studies. In addition, prodrug **13** was also selected for comparison purpose because it is a triglyceride-mimetic prodrug, an approach that was commonly used by other groups [16, 17].

### 3.5 Plasma pharmacokinetics and biodistribution of BEX and selected prodrugs

**Fig. 4:**
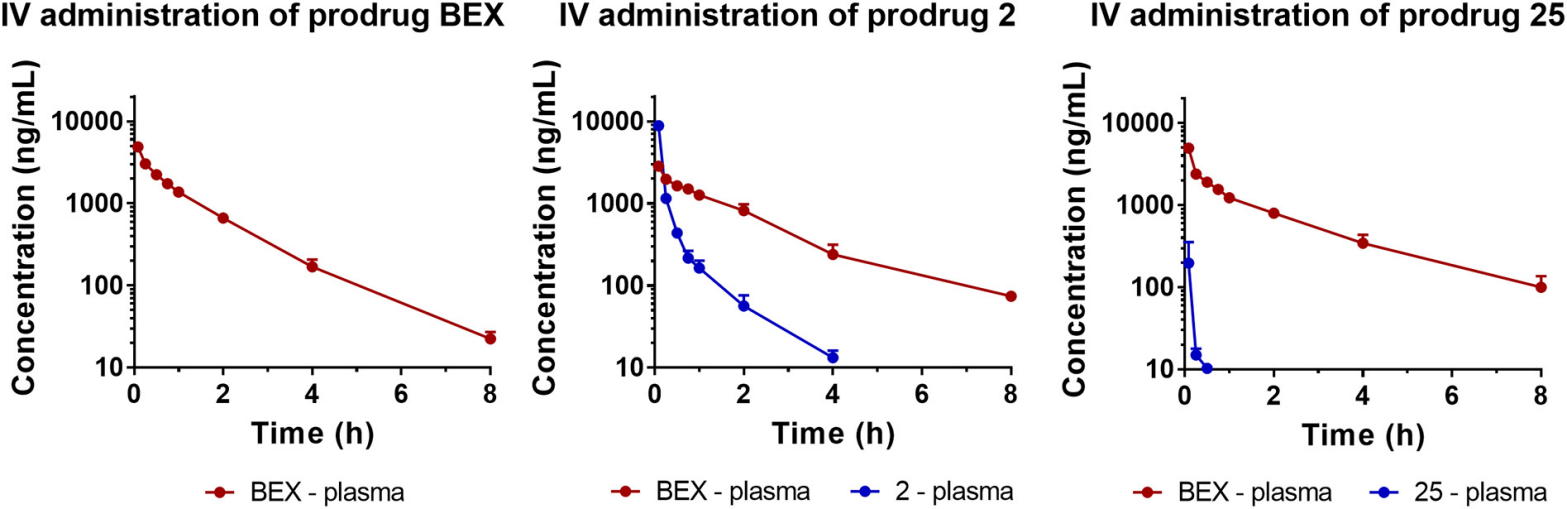
*In vivo* pharmacokinetic profiles of bexarotene (BEX) and its prodrugs following intravenous administration in rats. BEX or prodrugs **2** and **25** were administered at doses equivalent to 2.5 mg/kg of BEX (mean ± SD, *n* = 3 each). Pharmacokinetic profile of BEX following intravenous administration of BEX has been previously reported by our group [33] and is shown here for comparison.

**Fig. 5:**
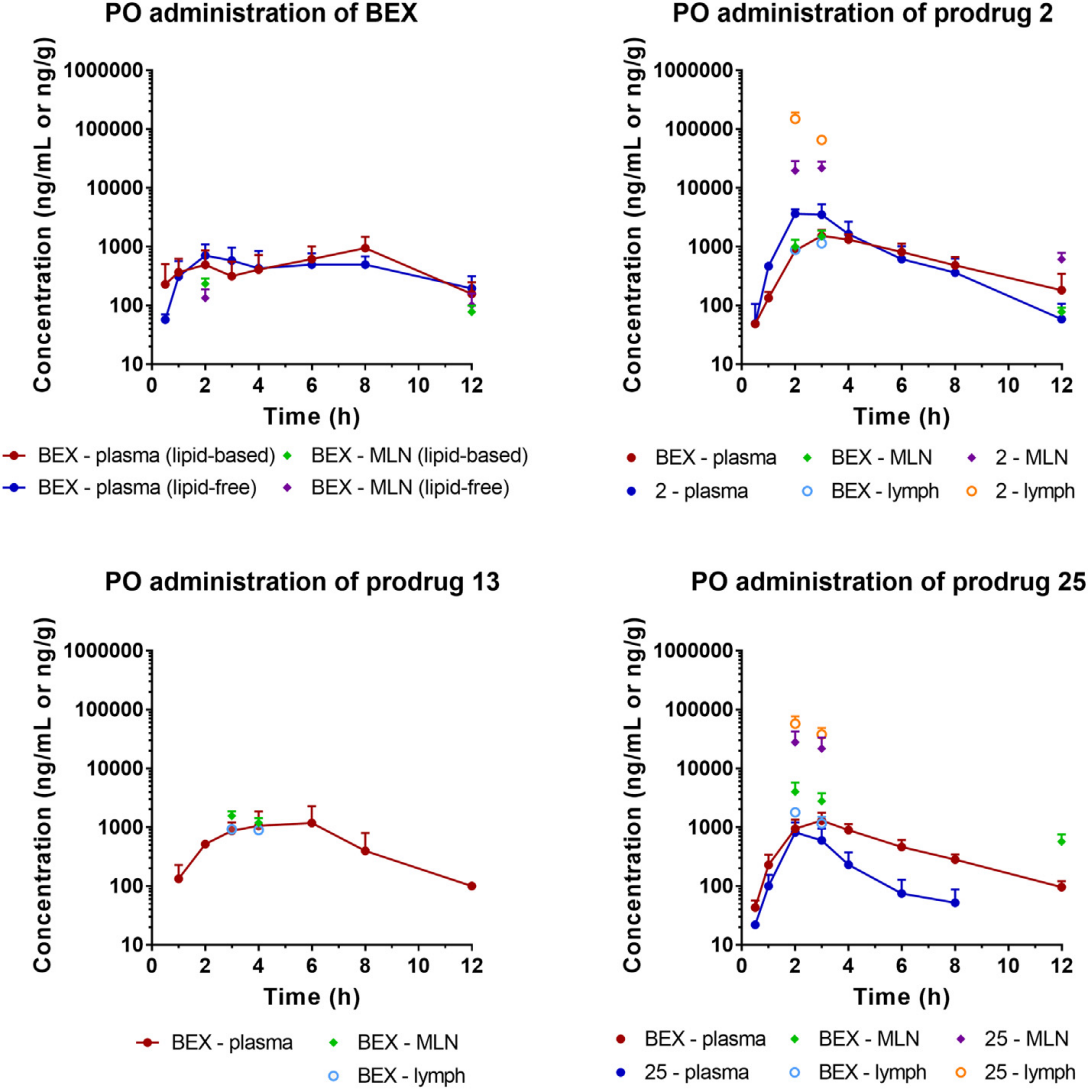
*In vivo* pharmacokinetic and biodistribution profiles of BEX and its prodrugs following oral administration in rats. BEX or prodrugs **2**, **13** and **25** were administered at doses equivalent to 10 mg/kg of BEX (mean ± SD, *n* = 5 for plasma and *n* = 4 for mesenteric lymph nodes (MLN) and lymph).

The *in vivo* pharmacokinetic and biodistribution profiles of BEX and its prodrugs were assessed following intravenous and oral administration in rats. When prodrugs were administered, the samples were analysed for concentrations of both the prodrug and BEX released from the prodrug. Fig. 4 shows plasma pharmacokinetic profiles following intravenous administration. Prodrug **2** was rapidly converted to BEX in plasma and there was no statistically significant difference between elimination half-lives (*t*_1/2_) of BEX following administration of BEX and the prodrug **2**. Fig. 5 shows plasma concentration-time profiles and biodistribution of BEX and its prodrugs following oral administration. Neither the plasma concentration-time profiles nor the pharmacokinetic parameters (Table 1) differed for BEX between lipid-free and lipid-coadministered groups following oral administration. The oral bioavailability values (*F*_*oral*_) of BEX following administration of BEX or each prodrug was calculated based on the intravenous administration profile of BEX obtained previously [33]. The bioavailabilities of BEX did not differ significantly between administration of BEX itself and administration of prodrugs (*p* > .05). The results of our study suggest that lymphatic transport has not changed the total amount of BEX absorbed but instead the drug absorbed into the enterocyte is re-routed to the intestinal lymphatics by association with CM. For assessment of biodistribution to the intestinal lymphatics following oral administration, the two sampling time points were considered as the most important for each compound in assessment of lymphatic transport: *t*_max_ observed in plasma and 1 h before *t*_max_. This was based on our previous reports showing that the concentration of drugs in the intestinal lymphatics could actually be higher prior to the *t*_max_ observed in plasma [5]. Additionally, tissues were harvested following the pharmacokinetic studies, which provided samples at 12 h time point.

**Table 1:** Plasma pharmacokinetic parameters of BEX following administration of BEX or its prodrugs by i.v. and p.o. administration (mean ± SD, n = 3 for i.v. and n = 5 for p.o.)

Compound dosed	BEX		2		25		13
Route of administration	p.o. (lipid-free)	p.o. (lipid-based)	i.v.	p.o.	i.v.	p.o.	p.o.
AUC_inf_ (h·ng/mL)	5836 ± 2140	6618 ± 1312	4614 ± 653	8645 ± 2598	5448 ± 301	5976 ± 1407	6887 ± 3821
AUC_0→t_ (h·ng/mL)	4907 ± 1787	5664 ± 1763	4443 ± 628	7751 ± 1552	5178 ± 182	5610 ± 1394	6538 ± 3956
C_0_ or C_max_ (ng/mL)	899 ± 338	1099 ± 432	2860 ± 189	1589 ± 360	4953 ± 614	1306 ± 464	1502 ± 1026
t_1/2_ (h)	3.0 ± 0.7	3.7 ± 1.2	1.6 ± 0.1	2.7 ± 1.0	1.8 ± 0.3	2.6 ± 0.4	2.5 ± 1.2
F_oral_ (%)^a^	26.5 ± 9.6	30.6 ± 9.5	–	41.8 ± 8.4	–	30.3 ± 7.5	35.3 ± 21.4

AUC_inf_, area under the curve from time zero to infinity; AUC_0➔t_, area under the curve from time zero to the last sampling time point; C_0_, concentration extrapolated to time zero; C_max_, maximum observed concentration; t_1/2_, half-life; F_oral_, oral bioavailability.

a Calculated based on the AUC_0➔t_ obtained following intravenous administration of BEX at 2.5 mg/kg [33].

Substantial delivery of prodrug **2** to the intestinal lymphatics was achieved (Fig. 5): the obtained concentrations were 41- and 6.0-fold higher in lymph and MLNs, respectively, compared to plasma. On the other hand, the concentrations of BEX released from prodrug **2** were comparable between the three matrices including MLNs, lymph and plasma. The results therefore demonstrate that the affinity of prodrug **2** to CM was sufficient to deliver considerable concentrations of the prodrug **2** to the intestinal lymphatics but the conversion rate was not high enough to yield high concentrations of BEX in the lymphatic system.

The *in vivo* assessment of prodrug **13** also showed no substantial intestinal lymphatic delivery of BEX (Fig. 5): the concentrations of BEX released from prodrug **13** in MLNs and lymph were found to be comparable to that in plasma. For prodrug **13**, only BEX was detected while the intact form of prodrug **13** was not found in any sample at any time point. This is consistent with a previous study which showed that TG-mimetic prodrugs are hydrolysed in the intestine before absorption and subsequent re-synthesis occurs in the enterocytes to yield TG-related molecules [16]. Conversion of prodrug **13** was not observed in rat plasma *in vitro*, but still *in vivo* it yielded levels of BEX in the intestinal lymphatics comparable to that in plasma (Fig. 5). This likely indicates that conversion of prodrug **13** to BEX would rely on lipase enzymes in the intestinal lumen (recognising the molecule as TG) rather than esterase-mediated release of BEX in plasma.

### 3.6 Development and assessment of activated ester prodrugs of BEX

Following the *in vivo* assessment results of prodrugs **2** and **13**, second generation activated ester prodrugs of BEX were designed and synthesised (**19–25**). It was thought that the release of BEX from the prodrugs need to be facilitated in order for the levels of BEX in the intestinal lymphatics to be significantly higher than in plasma. For the activated ester prodrugs, the recognition sites for the enzymes are away from the main scaffold thereby reducing steric hindrance of the enzymatic cleavage [34]. Indeed, most of these molecules showed higher rates of release of active drug (BEX) compared with the first generation prodrugs (Fig. 3b). Among these activated ester candidates, prodrug **25** was selected to proceed to *in vivo* studies due to its high affinity for CM (Fig. 3a) combined with the large difference between half-lives of active drug release in rat plasma and FaSSIF with esterase (Fig. 3b). Although prodrug **20** was also considered as a possible candidate, its low TG solubility (2.67 ± 0.06 mg/mL) precluded progress to *in vivo* studies. This is because the prodrugs needed to be formulated in lipid-based formulations which consist of mainly long-chain TG in order to stimulate the physiological process of intestinal lymphatic transport.

**Fig. 6:**
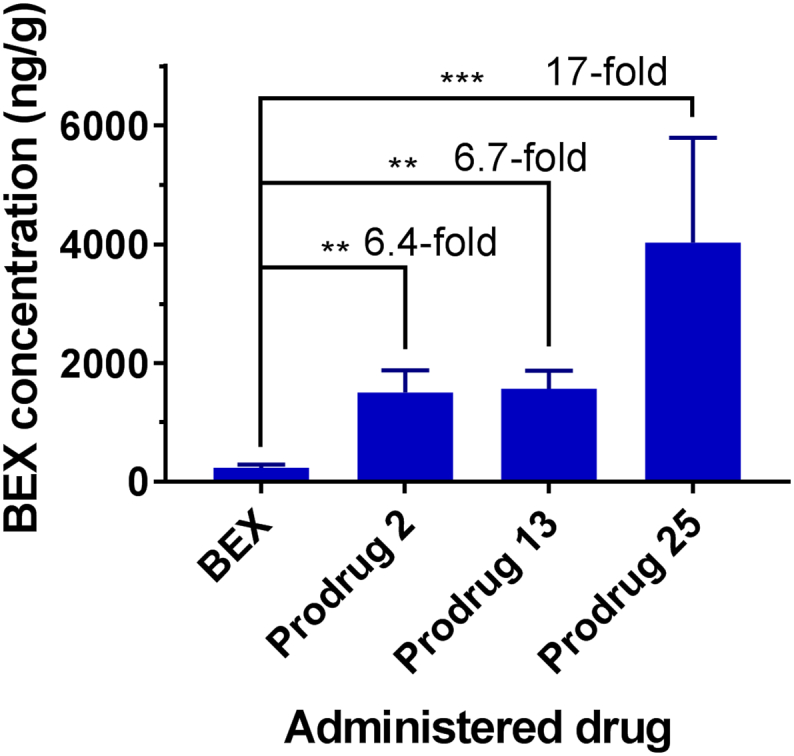
Maximum concentration of bexarotene (BEX) observed in mesenteric lymph nodes (ng/g) during biodistribution studies following oral administration of BEX or prodrugs 2, 13 and 25 (mean ± SD, *n* = 4). **, *p* < .01; ***, *p* < .001.

*In vivo* studies with prodrug **25** showed that it indeed released higher levels of free BEX in the intestinal lymphatic system compared to prodrugs **2** and **13** (Fig. 5). Although the concentrations of prodrug were still higher than those of BEX, the concentration of BEX achieved in MLNs was significantly higher than in plasma when prodrug **25** was administered (3.1-fold). In fact, this concentration of BEX in MLNs achieved by administration of prodrug **25** was 17-fold higher than that achieved by administration of BEX itself (Fig. 6). This result demonstrated that enhanced release of BEX from the activated ester prodrug significantly improved delivery of BEX to the MLNs.

### 3.7 Prodrug conversion in postprandial lymph

**Fig. 7:**
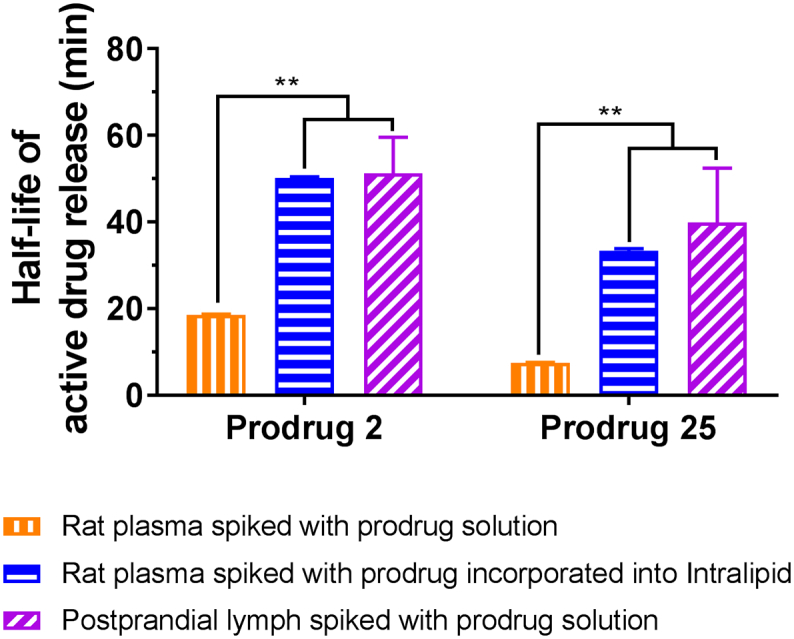
Detailed bio-relevant assessment of stability of prodrugs 2 and 25 mimicking various *in vivo* situations (mean ± SD, *n* = 3). Bars in orange vertical stripes represent stability of prodrugs when they are spiked in free form. Bars in blue horizontal stripes represent stability of prodrugs when they are spiked in the form of drug-chylomicron (CM) complex. Bars in purple diagonal stripes represent stability tested *ex vivo* with CM-rich postprandial lymph. **, *p* < .01. (For interpretation of the references to colour in this figure legend, the reader is referred to the web version of this article.)

It has been noted that there are differences between the release of BEX by the prodrugs in rat plasma *in vitro* and intestinal lymphatics *in vivo*. In rat plasma *in vitro*, the half-lives of active drug release of prodrugs **2** and **25** were 20 and 8 min, respectively, but contrary to this rapid conversion, the release of BEX in the intestinal lymphatics *in vivo* seemed to be slower. Therefore, it was thought that the release of BEX in the intestinal lymphatic system *in vivo* was slower than the release *in vitro* in rat plasma. We hypothesised that association of the prodrugs with lipoproteins could partially hinder their conversion, as the associated form would be passed on to the intestinal lymphatics. To test the hypothesis, we tested the stability of prodrugs in rat plasma, after incorporating them into lipid particles in the artificial emulsion prepared from Intralipid®. We indeed observed that emulsion-associated forms of the prodrugs exhibited delayed conversion in plasma (Fig. 7). We also collected postprandial lymph from rats and tested stability of these prodrugs. The half-lives of active drug release of prodrugs **2** and **25** obtained in postprandial lymph were prolonged similarly to the lipoprotein-associated forms in plasma, which could be due to association of the prodrugs with the very high number of CM present in the postprandial lymph (Fig. 7). This was further confirmed by the TG measurements in each test medium (Supplementary Material Table S1). Taken together, these results likely indicate that association with CM delays enzymatic conversion of the prodrugs in the intestinal lymphatics *in vivo* but does not prevent the release completely.

### 3.8 Application of the prodrug approach to intestinal lymphatic delivery of RA

**Fig. 8:**
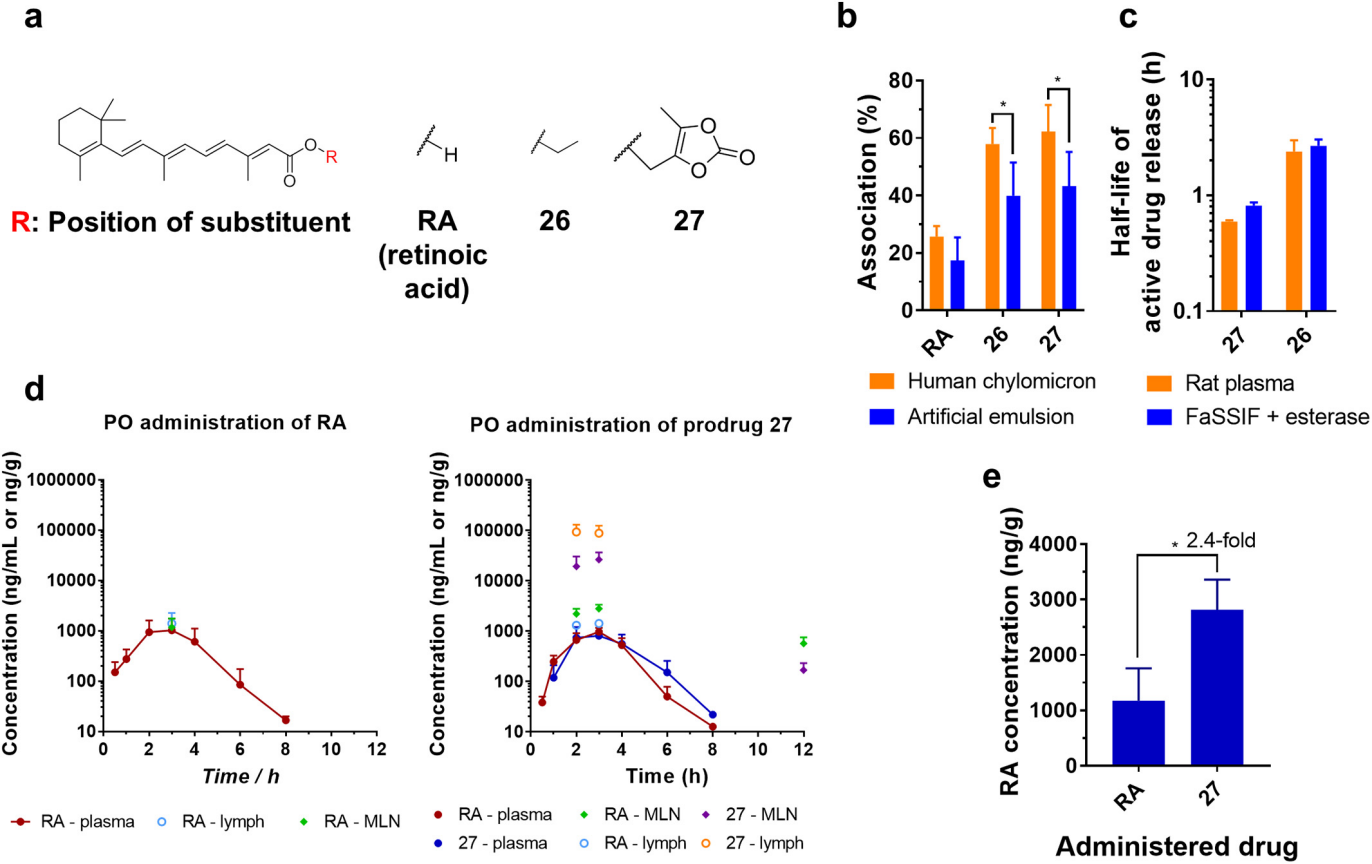
Application of the prodrug approach to RA (retinoic acid). (a) Chemical structures of RA and its prodrugs. (b) Association with human chylomicrons and artificial emulsion (mean ± SD, *n* = 6). (c) Half-life of active drug release in rat plasma and fasted state simulated intestinal fluid (FaSSIF) with esterase (mean ± SD, *n* = 3). (d) *In vivo* pharmacokinetic and biodistribution profiles in plasma and intestinal lymphatics following oral administration of RA (10 mg/kg) or prodrug 27 (at equivalent to 10 mg/kg RA) in rats (mean ± SD, *n* = 5 for plasma and *n* = 4 for mesenteric lymph nodes (MLN) and lymph). (e) Maximum concentration of RA observed in MLN (ng/g) during biodistribution studies following oral administration of RA and prodrug **27** (mean ± SD, n = 4). *, *p* < .05.

The prodrug approaches which resulted in successful intestinal lymphatic transport of BEX were applied to another drug, RA. Two lead prodrug candidates were synthesised: ethyl ester, **26** and cyclic ester, **27** (Fig. 8a). Both prodrugs **26** and **27** showed improved association with artificial emulsion and natural human CM compared with RA (Fig. 8b). The conversion rate of prodrug **27** to RA was faster than that of prodrug **26**, which was consistent with prodrugs **2** and **25** of BEX (Fig. 8c), and therefore prodrug **27** was selected as the lead RA prodrug for subsequent *in vivo* studies. RA and prodrug **27** were tested *in vivo* for plasma pharmacokinetics and biodistribution to the intestinal lymphatics following oral administration in conditions which facilitate lymphatic transport (Fig. 8d). The successful delivery of the prodrug **27** to the intestinal lymphatics and the release of RA resulted in 2.4-fold higher concentration of RA in MLNs compared to when RA itself was administered orally at equivalent doses (Fig. 8e). On the other hand, similar to the case of BEX and its prodrugs, the plasma AUCs of RA obtained following administration of RA and prodrug **27** did not differ significantly (3313 ± 1846 and 2541 ± 540 h·ng/mL, respectively, *p* > .05).

In addition, the anticancer effects of RA and BEX, separately and combined, were assessed by MTT assay using DLBCL cell lines. Significantly reduced GI_50_ values of BEX when it was co-administered with RA demonstrated improved potency and synergy of BEX and RA in DLBCL (Supplementary Material 2 and Supplementary Material Fig. S4).

## 4 Discussion

In this study the lipophilic ester prodrug approach was applied to deliver drugs selectively to the intestinal lymphatics. Systemic treatment usually struggles to deliver sufficiently high concentration of drugs into the intestinal lymphatics where many immune system-related diseases prevail [4, 7]. The concept underlying the lipophilic prodrug approach for efficient and selective lymphatic delivery was to take advantage of the physiological process of intestinal lymphatic absorption of dietary lipids from the GI tract. Lipophilic prodrugs of BEX had substantially higher association with CM compared with BEX, which was essential in this study as association with CM determines the extent of intestinal lymphatic transport [10]. The difference between log P and log D_7.4_, *i.e.* the effect of ionisation upon lipophilicity of compounds at pH 7.4, has previously been discussed as the most critical physicochemical property affecting the affinity to CM [20]. The BEX prodrugs studied here are carboxylic acid esters that are not ionisable (prior to hydrolysis), thus resulting in significantly improved association.

It should be noted that the lipophilic prodrug approach for delivery to intestinal lymphatics has been utilised in a limited number of previous studies [12]. However, much of the previous research employed TG-mimetic or long-chain fatty acid prodrug moieties in order to maximise lymphatic transport [8, 14, [16], [17], [18]]. In the current work we have also included TG-mimetic or long-chain fatty acid prodrug moieties (**10**−**13**), which indeed resulted in high association with CM (Fig. 3a and Supplementary Material Fig. S1). However, it was remarkable in our study that prodrugs with short-chain prodrug moieties (**1**, **2** and **4**) exhibited equally high affinity to CM, which indicated comparable potential for selective delivery to the intestinal lymphatics.

The stability of the prodrugs tested using rat plasma showed that hydrolysis of the ester bond was the main metabolic pathway. The decrease of prodrug concentration directly corresponded to the increase of active drug (BEX) concentration and no other metabolites of the prodrugs were observed in the chromatograms. A range of half-lives of active drug release was obtained from various prodrugs (Fig. 3b and Supplementary Material Fig. S3) which revealed structure-activity relationships: 1) the length and bulkiness of prodrugs correlated with prolonged half-lives, consistent with previous reports [35]; 2) comparison between prodrugs **2***vs.***3** and **15***vs.***16** showed that further substitution of the methylene group at position 1 of the prodrug moiety increased prodrug stability, also consistent with previous reports [36].

The plasma pharmacokinetic profiles and biodistribution of BEX to MLNs following oral administration of BEX itself were not affected by the presence of lipids and the concentrations in MLNs were lower than those in plasma (Fig. 5). This indicated that BEX itself had no intestinal lymphatic transport when it was administered orally in conditions which facilitate lymphatic absorption. Prodrug **2** had significantly higher association with CM than BEX and therefore resulted in substantially higher concentration in the intestinal lymphatics (MLNs and lymph) compared to plasma. Nevertheless, the release of BEX from the prodrug **2** was not sufficiently rapid so that the concentrations of BEX itself in the intestinal lymphatics were comparable to that in plasma. Findings following administration of prodrug **13** were similar to those of prodrug **2** and therefore we moved on to design second generation (activated ester) prodrugs which could potentially release the active drug at a higher conversion rate.

The activated ester prodrugs of BEX (**19**–**25**) were therefore designed to provide more extensive liberation of BEX. Unlike the first generation prodrugs (**1**–**18**), the ‘outer’ bond is recognised by the enzyme instead of the carboxyl ester bond which then subsequently leads to spontaneous liberation of the carboxylic acid [34]. Accordingly, these prodrugs could achieve faster conversion into BEX *in vitro* (Fig. 3b and Supplementary Material Fig. S3) and consequently higher release of BEX in the intestinal lymphatics *in vivo*, in the case of prodrug **25** (Fig. 5). As a result, the maximum concentration of BEX achieved in the MLNs following oral administration of prodrug **25** was 17-fold higher than that achieved following administration of BEX itself (Fig. 6). It should be emphasised that the MLNs are the physiological compartment of interest as this is where the relevant cancerous cells reside, HIV hides and replicates, and lymphadenopathy persists [[3], [4], [5], [6]].

The prodrug approach was additionally applied to RA. Similar to BEX, RA also possesses a carboxylic acid group and derivatisation of this group resulted in loss of ionisability of the compound, which enhanced association with CM (Fig. 8). The 2.4-fold increased concentration of RA achievable by administration of prodrug **27** demonstrated that the activated ester prodrug approach could be applied to also deliver RA selectively to the intestinal lymphatics. In addition, the synergistic anticancer effects of BEX and RA were demonstrated in DLBCL cell lines (Supplementary Material Fig. S4). BEX is a known specific agonist for retinoid X receptor (RXR) and all-*trans* RA is a specific agonist for the RA receptor (RAR) [37]. These receptors need to dimerise in order to exert their pharmacological effects and RXR can hetero-dimerise with RAR or peroxisome proliferator-activated receptor (PPAR) to regulate cell proliferation and differentiation [38]. Hetero-dimerisation between RXR-PPAR leads to cell proliferation whereas RXR-RAR hetero-dimers can evoke anti-proliferative effects and apoptosis [37]. The synergism between BEX and RA has not been reported previously and here we demonstrate that such synergism between the two drugs exists. Therefore, delivering both BEX and RA efficiently and selectively to the intestinal lymphatic system adopting an activated ester approach can potentially result in better treatment for people affected by DLBCL, especially when there is significant involvement of MLNs in the disease.

In this study, we report specific delivery of drugs to the intestinal lymphatics utilising the lipophilic prodrug approach. It had been previously stated that simple ester prodrugs would not be very efficient in enhancing intestinal lymphatic transport [8]. Indeed, lymphatic transport of BEX and RA prodrugs did not alter the overall bioavailability of the respective active drugs. However, our aim was to change the paradigm of intestinal lymphatic transport from increasing bioavailability to delivering high concentrations of the active drug to the intestinal lymphatic compartments. For this purpose, the suitable prodrugs proved to be ones that modify the physicochemical properties to increase the affinity to CM, while also readily releasing the active drug upon reaching the lymphatics. This was effectively accomplished by simple esterification, but required activated esters for enhanced release of the active moiety within the intestinal lymphatic system. The lack of change in the overall bioavailability of the active drug is in fact a substantial benefit for anticancer agents, as increased systemic exposure could lead to increased toxicity and side effects. It highlights the significance of prodrug **25**, since it increased exposure of the MLNs to BEX by 17-fold while maintaining the overall systemic bioavailability. It should be noted that in previous studies when TG mimetics or long chain fatty acids were used as prodrug approaches to deliver the drug into the intestinal lymphatics, the fold-increase in concentration achieved in MLNs was comparable to the prodrug **13** in this study, and substantially lower than what has been achieved by the activated ester approach (prodrug **25**) [16].

## 5 Conclusion

We herein report an activated ester lipophilic prodrug approach to deliver efficiently and selectively BEX and RA to the intestinal lymphatics. These two drugs have been demonstrated to exert synergistic growth inhibitory effects *in vitro* if delivered simultaneously. Prodrugs of a various range of physicochemical properties with different chain lengths and bulkiness were synthesised and assessed for delivery of the active drug to the intestinal lymphatics. The findings indicate that commonly used long-chain fatty acid prodrugs and TG mimetic prodrugs might not be the most efficient in delivering the active drug to the intestinal lymphatic system. However, we found that activated esters which promote conversion are very efficient for the purpose of delivery to and release of the active drug within the intestinal lymphatic system. The approach of activated ester prodrugs could benefit patients affected by a large number of pathological conditions with involvement of the intestinal lymphatics in the disease.

## Conflict of interest

The authors declare that they have no conflicts of interest.

## Appendix A Supplementary data

Supplementary material Image 1
