# Supplementary material for: Lipophilic activated ester prodrug approach for drug delivery to the intestinal lymphatic system
Source: J Control Release. 2018 Sep 28;286:10–9. doi: 10.1016/j.jconrel.2018.07.022 (PMC6143478; doi:10.1016/j.jconrel.2018.07.022)
Supplement: Supplementary file 1 — Supplementary material [file mmc1.docx]

**Supplementary Materials to:**

**Lipophilic activated ester prodrug approach for drug delivery to the intestinal lymphatic system**

Jong Bong Lee^1^, Atheer Zgair^1,2^, Jed Malec^1,3^, Tae Hwan Kim^4^, Min Gi Kim^5^, Joseph Ali^1^, Chaolong Qin^1^, Wanshan Feng^1^, Manting Chiang^6^, Xizhe Gao^6^, Gregory Voronin^7^, Aimie E. Garces^1^, Chun Long Lau^1^, Ting-Hoi Chan^1^, Amy Hume^1^, Tecashanell M. McIntosh^1^, Fadi Soukarieh^1^, Mohammed Al-Hayali^1^, Elena Cipolla^1,8^, Hilary M. Collins^1^, David M. Heery^1^, Beom Soo Shin^5^, Sun Dong Yoo^5^, Leonid Kagan^6^, Michael J. Stocks^1^, Tracey D. Bradshaw^1^, Peter M. Fischer^1^, Pavel Gershkovich^1,^*

^1^ School of Pharmacy, University of Nottingham, Nottingham, UK, NG7 2RD

^2^ College of Pharmacy, University of Anbar, Anbar, Iraq, 31001

^3^ DMPK, Evotec, Milton Park, Abingdon, Oxfordshire, UK, OX14 4RZ

^4^ College of Pharmacy, Catholic University of Daegu, Gyeongsan, Republic of Korea, 38430

^5^ School of Pharmacy, Sungkyunkwan University, Suwon, Republic of Korea, 16419

^6^ Department of Pharmaceutics, Ernest Mario School of Pharmacy, Rutgers, The State University of New Jersey, Piscataway, NJ, USA, 08854

^7^ Comparative Medicine Resources, Rutgers, The State University of New Jersey, Piscataway, NJ, USA, 08854

^8^ School of Pharmacy, Universita di Roma Tor Vergata, Rome, Italy, 00173

***Corresponding author:** Pavel Gershkovich, PhD

School of Pharmacy, Centre for Biomolecular Sciences

University of Nottingham, University Park

Nottingham, UK

NG7 2RD

Tel:  +44 (0) 115 846 8014

Fax: +44 (0) 115 951 3412

Email: [pavel.gershkovich@nottingham.ac.uk](mailto:pavel.gershkovich@nottingham.ac.uk)

**Supplementary Material 1. Synthesis and characterisation of prodrugs**

^1^H NMR and ^13^C NMR spectra were obtained using Bruker 400 Ultrashield Spectrometer at 400 and 100 MHz, respectively. Bruker TOPSPIN 2.1 software was used to analyse the spectra. Chemical shifts were reported as parts per million (ppm) relative to the tetramethylsilane (internal standard) to the nearest 0.01 ppm and coupling constants (*J*) to the nearest 0.1 Hz. The following abbreviations were used while quoting the spectra: s, singlet; d, doublet; t, triplet; q, quartet; m, multiplet. High resolution mass spectrometry (HR-MS) was performed using Bruker MicroTOF operated with electrospray ionisation in positive mode. Purity of each compound was tested in respective chromatographic conditions as stated in Supplementary Table S2.

**1 (bexarotene methyl ester; methyl 4-(1-(3,5,5,8,8-pentamethyl-5,6,7,8-tetrahydronaphthalen-2-yl)vinyl)benzoate).** General synthetic scheme for esterification of BEX was used with methanol as the reactant. The final compound was obtained as a white solid (82.0% yield), melting point 150-152 °C. Purity was >98% by HPLC-UV. ^1^H NMR (400 MHz, CDCl_3_): δ 7.98 (dt, *J_1_* = 8.4 Hz, *J_2_* =1.8 Hz, 2H), 7.37 (dt, *J_1_* = 8.4 Hz, *J_2_* = 1.8 Hz, 2H), 7.15 (s, 1H), 7.10 (s, 1H), 5.83 (d, *J* = 1.2 Hz, 1H), 5.34 (d, *J* = 1.2 Hz, 1H), 3.93 (s, 3H), 1.97 (s, 3H), 1.73 (s, 4H), 1.33 (s, 6H), 1.30 (s, 6H). ^13^C NMR (100 MHz, CDCl_3_): δ 167.12, 149.35, 145.74, 144.52, 142.48, 138.18, 132.88, 129.78, 129.13, 128.21, 128.17, 126.72, 116.94, 52.18, 35.36, 34.14, 34.03, 32.07, 32.03, 22.76, 20.07. HR-MS (ESI^+^): *m/z* [M+ H]^+^ calculated for C_25_H_31_O_2_, 363.2319, found 363.2306.

**2 (bexarotene ethyl ester; ethyl 4-(1-(3,5,5,8,8-pentamethyl-5,6,7,8-tetrahydronaphthalen-2-yl)vinyl)benzoate).** General synthetic scheme for esterification of BEX was used with ethanol as the reactant. The final compound was obtained as white crystal (66.9% yield), melting point 95-97 °C. Purity was >97% by HPLC-UV. ^1^H NMR (400 MHz, CDCl_3_): δ 7.98 (dt, *J_1_* = 8.5 Hz, *J_2_* =1.9 Hz, 2H), 7.35 (dt, *J_1_* = 8.5 Hz, *J_2_* = 1.9 Hz, 2H), 7.14 (s, 1H), 7.09 (s, 1H), 5.82 (d, *J* = 1.3 Hz, 1H), 5.34 (d, *J* = 1.3 Hz, 1H), 4.39 (q, *J* = 7.2 Hz, 2H), 1.96 (s, 3H), 1.72 (s, 4H), 1.40 (t, *J* = 7.1 Hz, 3H), 1.32 (s, 6H), 1.29 (s, 6H). ^13^C NMR (100 MHz, CDCl_3_): δ 166.63, 149.36, 145.61, 144.46, 142.44, 138.21, 132.86, 129.74, 129.48, 128.20, 128.17, 126.67, 116.81, 60.99, 35.36, 34.14, 34.03, 32.07, 32.03, 20.06, 14.48. HR-MS (ESI^+^): *m/z* [M+ H]^+^ calculated for C_26_H_33_O_2_, 377.2475, found 377.2470.

**3 (bexarotene isopropyl ester; isopropyl 4-(1-(3,5,5,8,8-pentamethyl-5,6,7,8-tetrahydronaphthalen-2-yl)vinyl)benzoate).** General synthetic scheme for esterification of BEX was used with isopropanol as the reactant. The final compound was obtained as white solid (45.0% yield), melting point 77-79 °C. Purity was >98% by HPLC-UV.  ^1^H NMR (400 MHz, CDCl_3_): δ 7.98 (dt, *J_1_* = 8.6 Hz, *J_2_* =1.8 Hz, 2H), 7.35 (dt, *J_1_* = 8.6 Hz, *J_2_* = 1.8 Hz, 2H), 7.15 (s, 1H), 7.10 (s, 1H), 5.82 (d, *J* = 1.4 Hz, 1H), 5.34 (d, *J* = 1.3 Hz, 1H), 5.26 (septet, *J* = 6.2 Hz, 1H), 1.96 (s, 3H), 1.72(s, 4H), 1.38 (d, *J* = 6.2 Hz, 6H), 1.33 (s, 6H), 1.30 (s, 6H). ^13^C NMR (100 MHz, CDCl_3_): δ 166.11, 149.38, 145.50, 144.45, 142.43, 138.23, 132.86, 129.88, 129.69, 128.18, 128.15, 126.61, 116.71, 68.37, 35.37, 35.35, 34.13, 34.03, 32.07, 32.02, 22.09, 20.04. HR-MS (ESI^+^): *m/z* [M+ H]^+^ calculated for C_27_H_35_O_2_, 391.2632, found 391.2636.

**4 (bexarotene propyl ester; propyl 4-(1-(3,5,5,8,8-pentamethyl-5,6,7,8-tetrahydronaphthalen-2-yl)vinyl)benzoate).** General synthetic scheme for esterification of BEX was used with 1-propanol as the reactant. The final compound was obtained as white solid (90.0% yield), melting point 66-68 °C. Purity was 98.0% by HPLC-UV. ^1^H NMR (400 MHz, CDCl_3_): δ 8.00 (dt, *J_1_* = 8.4 Hz, *J_2_* =1.9 Hz, 2H), 7.37 (dt, *J_1_* = 8.4 Hz, *J_2_* = 1.9 Hz, 2H), 7.16 (s, 1H), 7.11 (s, 1H), 5.84 (d, *J* = 1.1 Hz, 1H), 5.35 (d, *J* = 1.1 Hz, 1H), 4.30 (t, *J* = 6.7 Hz, 2H), 1.98 (s, 3H), 1.81 (sextet, *J* = 7.2 Hz, 2H), 1.73 (s, 4H), 1.34 (s, 6H), 1.31 (s, 6H) 1.05 (t, *J* = 7.4 Hz, 3H). ^13^C NMR (100 MHz, CDCl_3_): δ 166.69, 149.38, 145.61, 144.47, 142.45, 138.22, 132.87, 129.75, 129.50, 128.21, 128.17, 126.68, 116.80, 66.60, 41.51, 36.23, 35.38, 35.36, 34.14, 34.04, 33.88, 32.08, 32.03, 29.82, 29.52, 29.22, 29.04, 27.83, 22.85, 22.81, 22.76, 22.28, 20.60, 20.06, 19.58, 18.91, 14.46, 14.26, 11.57, 10.66. HR-MS (ESI^+^): *m/z* [M+ H]^+^ calculated for C_27_H_35_O_2_, 391.2632, found 391.2636.

**5 (bexarotene butyl ester; butyl 4-(1-(3,5,5,8,8-pentamethyl-5,6,7,8-tetrahydronaphthalen-2-yl)vinyl)benzoate).** General synthetic scheme for esterification of BEX was used with 1-butanol as the reactant. The final compound was obtained as white solid (102 mg, 87.8% yield), melting point 54-56 °C. Purity was >98% by HPLC-UV. ^1^H NMR (400 MHz, CDCl_3_): δ 7.99 (dt, *J_1_* = 8.4 Hz, *J_2_* =1.9 Hz, 2H), 7.37 (dt, *J_1_* = 8.4 Hz, *J_2_* = 1.9 Hz, 2H), 7.15 (s, 1H), 7.10 (s, 1H), 5.83 (d, *J* = 1.2 Hz, 1H), 5.34 (d, *J* = 1.2 Hz, 1H), 4.34 (t, *J* = 6.6 Hz, 2H), 1.97 (s, 3H), 1.77 (quintet, *J* = 8.0 Hz, 2H), 1.73 (s, 4H), 1.50 (sextet, *J* = 7.6 Hz, 2H), 1.33 (s, 6H), 1.30 (s, 6H), 1.00 (t, *J* = 7.4 Hz, 3H). ^13^C NMR (100 MHz, CDCl_3_): δ 166.70, 149.36, 145.60, 144.47, 142.44, 138.21, 132.86, 129.74, 129.49, 128.20, 128.16, 126.67, 116.80, 64.89, 41.50, 35.37, 35.35, 34.14, 34.03, 32.07, 32.03, 30.94, 29.85, 29.81, 29.51, 29.21, 29.04, 27.82, 22.76, 22.48, 20.06, 19.58, 19.43, 14.46, 14.26, 14.20, 13.90, 11.57. HR-MS (ESI^+^): *m/z* [M+ H]^+^ calculated for C_28_H_37_O_2_, 405.2788, found 405.2777.

**6 (bexarotene isobutyl ester; isobutyl 4-(1-(3,5,5,8,8-pentamethyl-5,6,7,8-tetrahydronaphthalen-2-yl)vinyl)benzoate).** General synthetic scheme for esterification of BEX was used with isobutanol as the reactant. The final compound was obtained as white solid (89.5% yield), melting point 62-64 °C. Purity was >97% by HPLC-UV. ^1^H NMR (400 MHz, CDCl_3_): δ 7.95 (dt, *J_1_* = 8.5 Hz, *J_2_* =1.8 Hz, 2H), 7.34 (dt, *J_1_* = 8.5 Hz, *J_2_* = 1.8 Hz, 2H), 7.12 (s, 1H), 7.08 (s, 1H), 5.81 (d, *J* = 1.3 Hz, 1H), 5.33 (d, *J* = 1.3 Hz, 1H), 4.47 (d, *J* = 6.3 Hz, 2H), 2.10 (nonet, *J* = 6.7 Hz, 1H), 1.98 (s, 3H), 1.73 (s, 4H), 1.34 (s, 6H), 1.31 (s, 6H), 1.05 (d, *J* = 6.8 Hz, 6H). ^13^C NMR (100 MHz, CDCl_3_): δ 166.63, 149.37, 145.61, 144.46, 142.45, 138.22, 132.85, 129.74, 129.49, 128.19, 128.16, 126.67, 116.78, 71.07, 41.50, 35.38, 35.36, 34.13, 34.03, 32.07, 32.03, 29.20, 28.07, 27.81, 22.75, 20.59, 20.04, 19.57, 19.32, 11.56. HR-MS (ESI^+^): *m/z* [M+ H]^+^ calculated for C_28_H_37_O_2_, 405.2788, found 405.2779.

**7 (bexarotene 3,3’-dihydroxyisobutyl ester; 3-hydroxy-2-(hydroxymethyl)propyl 4-(1-(3,5,5,8,8-pentamethyl-5,6,7,8-tetrahydronaphthalen-2-yl)vinyl)benzoate).** General synthetic scheme for esterification of BEX was used with 2-hydroxymethyl-1,3-propanediol and *N,N*-dimethylformamide (DMF) as the reactant and the organic solvent, respectively. The flash chromatography for purification was also different from the general synthetic scheme that the following solvents were used for mobile phase: hexane:ethyl acetate = 50:50, 500 mL; hexane:ethyl acetate = 40:60, 250 mL; hexane:ethyl acetate = 30:70, 250 mL; hexane:ethyl acetate = 20:80, 500 mL. The final compound was obtained as white crystal (56.6% yield), melting point 116-118 °C. Purity was >98% by HPLC-UV. ^1^H NMR (400 MHz, CDCl_3_): δ 7.95 (dt, *J_1_* = 8.5 Hz, *J_2_* =1.8 Hz, 2H), 7.34 (dt, *J_1_* = 8.5 Hz, *J_2_* = 1.8 Hz, 2H), 7.12 (s, 1H), 7.08 (s, 1H), 5.81 (d, *J* = 1.3 Hz, 1H), 5.33 (d, *J* = 1.3 Hz, 1H), 4.47 (d, *J* = 6.3 Hz, 2H), 3.93-3.76 (m, 4H), 3.13 (broad s, 2H), 2.18 (septet, *J* = 5.5 Hz, 1H), 1.94 (s, 3H), 1.70 (s, 4H), 1.30 (s, 6H), 1.28 (s, 6H). ^13^C NMR (100 MHz, CDCl_3_): δ 166.97, 149.19, 145.93, 144.47, 142.43, 138.04, 132.76, 129.80, 128.79, 128.12, 126.70, 116.98, 62.97, 62.32, 42.71, 35.30, 35.28, 34.08, 33.97, 32.01, 31.96, 19.99. HR-MS (ESI^+^): *m/z* [M+ H]^+^ calculated for C_28_H_37_O_4_, 437.2686, found 437.2680.

**8 (bexarotene hexyl ester; hexyl 4-(1-(3,5,5,8,8-pentamethyl-5,6,7,8-tetrahydronaphthalen-2-yl)vinyl)benzoate).** General synthetic scheme for esterification of BEX was used with 1-hexanol as the reactant. The final compound was obtained as white solid (91.8% yield), melting point 46-48 °C. Purity was >98% by HPLC-UV. ^1^H NMR (400 MHz, CDCl_3_): δ 7.99 (dt, *J_1_* = 8.5 Hz, *J_2_* =1.8 Hz, 2H), 7.37 (dt, *J_1_* = 8.5 Hz, *J_2_* = 1.8 Hz, 2H), 7.16 (s, 1H), 7.11 (s, 1H), 5.84 (d, *J* = 1.3 Hz, 1H), 5.35 (d, *J* = 1.3 Hz, 1H), 4.33 (t, *J* = 6.7 Hz, 2H), 1.97 (s, 3H), 1.78 (quintet, *J* = 7.9 Hz, 2H), 1.73 (s, 4H), 1.47 (quintet, *J* = 7.2 Hz, 2H), 1.40-1.34 (m, 4H), 1.33 (s, 6H), 1.31 (s, 6H), 0.93 (t, *J* = 7.0 Hz, 3H). ^13^C NMR (100 MHz, CDCl_3_): δ 166.72, 149.36, 145.60, 144.47, 142.45, 138.21, 132.87, 129.74, 129.51, 128.20, 128.16, 126.67, 116.82, 65.21, 35.37, 35.36, 34.14, 34.04, 32.08, 32.03, 31.62, 28.86, 25.87, 22.70, 20.06, 14.15. HR-MS (ESI^+^): *m/z* [M+ H]^+^ calculated for C_30_H_41_O_2_, 433.3101, found 433.3097.

**9 (bexarotene octyl ester; octyl 4-(1-(3,5,5,8,8-pentamethyl-5,6,7,8-tetrahydronaphthalen-2-yl)vinyl)benzoate).** General synthetic scheme for esterification of BEX was used with 1-octanol as the reactant. The final compound was obtained as white solid (87.1% yield), melting point 66-68°C. Purity was >98% by HPLC-UV. ^1^H NMR (400 MHz, CDCl_3_): δ 7.99 (dt, *J_1_* = 8.6 Hz, *J_2_* =1.8 Hz, 2H), 7.37 (dt, *J_1_* = 8.6 Hz, *J_2_* = 1.8 Hz, 2H), 7.16 (s, 1H), 7.11 (s, 1H), 5.84 (d, *J* = 1.4 Hz, 1H), 5.35 (d, *J* = 1.3 Hz, 1H), 4.33 (t, *J* = 6.7 Hz, 2H), 1.97 (s, 3H), 1.78 (quintet, *J* = 6.7 Hz, 2H), 1.73 (s, 4H), 1.51-1.42 (m, 2H), 1.40-1.26 (m, 20H), 0.91 (t, *J* = 7.0 Hz, 3H), 1.47 (quintet, *J* = 7.2 Hz, 2H), 1.40-1.34 (m, 4H), 1.32 (d, *J* = 11.2 Hz, 12H), 0.91 (t, *J* = 7.0 Hz, 3H). ^13^C NMR (100 MHz, CDCl_3_): δ 166.69, 149.36, 145.59, 144.46, 142.44, 138.22, 132.86, 129.74, 129.51, 128.20, 128.16, 126.67, 116.80, 65.20, 41.50, 35.37, 35.35, 34.13, 34.03, 32.07, 32.03, 31.94, 29.40, 29.34, 29.21, 29.04, 28.89, 26.21, 22.84, 22.79, 22.77, 20.59, 20.06, 19.58, 14.46, 14.23, 11.57. HR-MS (ESI^+^): *m/z* [M+ H]^+^ calculated for C_32_H_45_O_2_, 461.3414, found 461.3392.

**10 (bexarotene undecanyl ester; undecyl 4-(1-(3,5,5,8,8-pentamethyl-5,6,7,8-tetrahydronaphthalen-2-yl)vinyl)benzoate).** General synthetic scheme for esterification of BEX was used with 1-undecanol as the reactant. The final compound was obtained as white solid (70.0% yield), melting point 38-40 °C. Purity was >98% by HPLC-UV. ^1^H NMR (400 MHz, CDCl_3_): δ 7.98 (dt, *J_1_* = 8.6 Hz, *J_2_* =1.8 Hz, 2H), 7.36 (dt, *J_1_* = 8.6 Hz, *J_2_* = 1.8 Hz, 2H), 7.15 (s, 1H), 7.09 (s, 1H), 5.83 (d, *J* = 1.4 Hz, 1H), 5.34 (d, *J* = 1.3 Hz, 1H), 4.32 (t, *J* = 6.6 Hz, 2H), 1.96 (s, 3H), 1.77 (quintet, *J* = 6.6 Hz, 2H), 1.72 (s, 4H), 1.50-1.41 (m, 2H), 1.41-1.20 (m, 26H), 0.90 (t, *J* = 6.6 Hz, 3H). ^13^C NMR (100 MHz, CDCl_3_): δ 166.71, 149.38, 145.60, 144.48, 142.46, 138.22, 132.87, 129.74, 129.52, 128.21, 128.17, 126.67, 116.80, 65.22, 35.38, 35.37, 34.15, 34.04, 32.08, 32.05, 32.04, 29.74, 29.73, 29.68, 29.47, 29.44, 29.90, 26.21, 22.83, 20.06, 14.25. HR-MS (ESI^+^): *m/z* [M+ H]^+^ calculated for C_35_H_51_O_2_, 503.3884, found 503.3884.

**11 (bexarotene oleyl ester; (E)-octadec-9-en-1-yl 4-(1-(3,5,5,8,8-pentamethyl-5,6,7,8-tetrahydronaphthalen-2-yl)vinyl)benzoate).** General synthetic scheme for esterification of BEX was used with oleyl alcohol as the reactant. The final compound was obtained as colourless gum (90.7% yield). Purity was >98% by HPLC-UV. ^1^H NMR (400 MHz, CDCl_3_): δ 7.99 (dt, *J_1_* = 8.5 Hz, *J_2_* =1.8 Hz, 2H), 7.37 (dt, *J_1_* = 8.5 Hz, *J_2_* = 1.8 Hz, 2H), 7.16 (s, 1H), 7.11 (s, 1H), 5.84 (d, *J* = 1.3 Hz, 1H), 5.38 (tt, *J_1_* = 5.5 Hz, *J_2_* = 1.8 Hz, 2H), 5.35 (d, *J* = 1.3 Hz, 1H), 4.33 (t, *J* = 6.6 Hz, 2H), 2.09-2.00 (m, 4H), 1.97 (s, 3H), 1.78 (quintet, *J* = 7.0 Hz, 2H), 1.73 (s, 4H), 1.51-1.42 (m, 2H), 1.42-1.21 (m, 32H), 0.91 (t, *J* = 6.7 Hz, 3H). ^13^C NMR (100 MHz, CDCl_3_): δ 166.67, 149.37, 145.59, 144.46, 142.44, 138.22, 132.86, 130.12, 129.93, 129.74, 129.51, 128.19, 128.16, 126.66, 116.78, 65.17, 35.38, 35.36, 34.14, 34.03, 32.07, 32.05, 32.03, 29.92, 29.88, 29.67, 29.58, 29.46, 29.42, 29.36, 28.90, 27.36, 27.33, 26.20, 22.83, 20.05, 14.25. HR-MS (ESI^+^): *m/z* [M+ H]^+^ calculated for C_42_H_63_O_2_, 599.4823, found 599.4828.

**12 (bexarotene octadecanyl ester; octadecyl 4-(1-(3,5,5,8,8-pentamethyl-5,6,7,8-tetrahydronaphthalen-2-yl)vinyl)benzoate).** General synthetic scheme for esterification of BEX was used with 1-octadecanol as the reactant. The final compound was obtained as white solid (67.9% yield), melting point 62-64 °C. Purity was >98% by HPLC-UV. ^1^H NMR (400 MHz, CDCl_3_): δ 7.99 (dt, *J_1_* = 8.5 Hz, *J_2_* =1.8 Hz, 2H), 7.37 (dt, *J_1_* = 8.5 Hz, *J_2_* = 1.8 Hz, 2H), 7.16 (s, 1H), 7.10 (s, 1H), 5.83 (d, *J* = 1.3 Hz, 1H), 5.35 (d, *J* = 1.2 Hz, 1H), 4.33 (t, *J* = 6.6 Hz, 2H), 1.97 (s, 3H), 1.78 (quintet, *J* = 6.7 Hz, 2H), 1.73 (s, 4H), 1.51-1.42 (m, 2H), 1.42-1.20 (m, 40H), 0.91 (t, *J* = 6.6 Hz, 3H). ^13^C NMR (100 MHz, CDCl_3_): δ 166.69, 149.38, 145.59, 144.46, 142.45, 138.22, 132.86, 129.74, 129.52, 128.29, 128.16, 126.67, 116.79, 65.20, 35.38, 35.37, 34.14, 34.04, 32.08, 32.03, 29.85, 29.82, 29.74, 29.70, 29.51, 29.45, 28.91, 26.22, 22.84, 20.06, 14.26. HR-MS (ESI^+^): *m/z* [M+ H]^+^ calculated for C_42_H_65_O_2_, 601.4979, found 601.4943.

**13 (bexarotene triglyceryl ester; 2-((4-(1-(3,5,5,8,8-pentamethyl-5,6,7,8-tetrahydronaphthalen-2-yl)vinyl)benzoyl)oxy)propane-1,3-diyl dipalmitate).** General synthetic scheme for esterification of BEX was used with glyceryl 1,3-dipalmitate as the reactant. The final compound was obtained as white solid (60.4% yield), melting point 46-48°C. Purity was >98% by HPLC-UV. ^1^H NMR (400 MHz, CDCl_3_): δ 7.97 (dt, *J_1_* = 8.5 Hz, *J_2_* =1.7 Hz, 2H), 7.37 (dt, *J_1_* = 8.5 Hz, *J_2_* = 1.7 Hz, 2H), 7.14 (s, 1H), 7.10 (s, 1H), 5.84 (d, *J* = 1.3 Hz, 1H), 5.52 (quintet, *J* = 4.4 Hz, 1H), 5.36 (d, *J* = 1.3 Hz, 1H), 4.44-4.30 (m, 4H), 2.33 (t, *J* = 7.4 Hz, 4H), 1.97 (s, 3H), 1.73 (s, 4H), 1.61 (quintet, *J* = 7.2 Hz, 4H), 1.37-1.21 (m, 60H), 0.91 (t, *J* = 6.6 Hz, 6H). ^13^C NMR (100 MHz, CDCl_3_): δ 173.46, 165.61, 149.25, 146.13, 144.54, 142.49, 138.09, 132.81, 130.01, 128.49, 128.18, 126.74, 117.06, 69.86, 62.28, 35.35, 34.22, 34.14, 34.03, 32.07, 32.02, 29.84, 29.80, 29.75, 29.57, 29.50, 29.37, 29.23, 25.01, 22.83, 22.75, 20.05, 14.25.

**14 (bexarotene 2-ethylbutyl ester; 2-ethylbutyl 4-(1-(3,5,5,8,8-pentamethyl-5,6,7,8-tetrahydronaphthalen-2-yl)vinyl)benzoate).** General synthetic scheme for esterification of BEX was used with 2-ethyl-1-butanol as the reactant. The final compound was obtained as colourless oil (90.5% yield). Purity was >98% by HPLC-UV. ^1^H NMR (400 MHz, CDCl_3_): δ 7.99 (dt, *J_1_* = 8.5 Hz, *J_2_* =1.8 Hz, 2H), 7.37 (dt, *J_1_* = 8.5 Hz, *J_2_* = 1.8 Hz, 2H), 7.16 (s, 1H), 7.10 (s, 1H), 5.84 (d, *J* = 1.3 Hz, 1H), 5.35 (d, *J* = 1.3 Hz, 1H), 4.27 (d, *J* = 5.7 Hz, 2H), 1.97 (s, 3H), 1.73 (s, 4H), 1.68 (septet, *J* = 6.2 Hz, 1H), 1.48 (quintet, *J* = 7.2 Hz, 4H), 1.33 (s, 6H), 1.31 (s, 6H), 0.97 (t, *J* = 7.4 Hz, 6H). ^13^C NMR (100 MHz, CDCl_3_): δ 166.74, 149.37, 145.59, 144.46, 142.45, 138.22, 132.86, 129.74, 129.54, 128.19, 128.16, 126.67, 116.78, 67.01, 41.51, 40.71, 36.52, 36.23, 35.38, 35.36, 34.28, 34.14, 34.04, 33.88, 32.08, 32.03, 29.21, 29.04, 27.82, 25.98, 23.70, 22.76, 22.48, 20.59, 20.05, 19.58, 18.91, 14.46, 14.20, 11.57, 11.28. HR-MS (ESI^+^): *m/z* [M+ H]^+^ calculated for C_30_H_41_O_2_, 433.3101, found 433.3094.

**15 (bexarotene benzyl ester; benzyl 4-(1-(3,5,5,8,8-pentamethyl-5,6,7,8-tetrahydronaphthalen-2-yl)vinyl)benzoate).** General synthetic scheme for esterification of BEX was used with benzyl alcohol as the reactant. The final compound was obtained as colourless oil (83.4% yield). Purity was >98% by HPLC-UV. ^1^H NMR (400 MHz, CDCl_3_): δ 8.03 (dt, *J_1_* = 8.5 Hz, *J_2_* =1.6 Hz, 2H), 7.50-7.33 (m, 7H), 7.16 (s, 1H), 7.11 (s, 1H), 5.84 (d, *J* = 1.2 Hz, 1H), 5.39 (s, 2H), 5.36 (d, *J* = 1.2 Hz, 1H), 1.97 (s, 3H), 1.73 (s, 4H), 1.34 (s, 6H), 1.31 (s, 6H). ^13^C NMR (100 MHz, CDCl_3_): δ 166.42, 149.32, 145.85, 144.49, 142.46, 138.16, 136.29, 132.85, 129.92, 129.09, 128.72, 128.33, 128.24, 128.19, 128.18, 126.72, 116.92, 66.73, 41.50, 36.23, 35.67, 35.35, 34.27, 34.14, 34.03, 33.87, 32.07, 32.03, 29.21, 29.04, 27.82, 25.98, 22.76, 22.48, 20.59, 20.05, 19.58, 18.91, 14.46, 14.20, 11.57, 9.00. HR-MS (ESI^+^): *m/z* [M+ H]^+^ calculated for C_31_H_35_O_2_, 439.2632, found 439.2626.

**16 (bexarotene 1-phenylethyl ester; 1-phenylethyl 4-(1-(3,5,5,8,8-pentamethyl-5,6,7,8-tetrahydronaphthalen-2-yl)vinyl)benzoate).** General synthetic scheme for esterification of BEX was used with 1-phenylethanol as the reactant. The final compound was obtained as colourless oil (74.1% yield). Purity was >98% by HPLC-UV. ^1^H NMR (400 MHz, CDCl_3_): δ 8.03 (dt, *J_1_* = 8.5 Hz, *J_2_* =1.8 Hz, 2H), 7.51-7.29 (m, 7H), 7.16 (s, 1H), 7.11 (s, 1H), 6.15 (quartet, *J* = 6.6 Hz, 1H), 5.84 (d, *J* = 1.2 Hz, 1H), 5.35 (d, *J* = 1.2 Hz, 1H), 1.97 (s, 3H), 1.73 (s, 4H), 1.69 (d, *J* = 6.6 Hz, 3H), 1.34 (s, 6H), 1.31 (s, 6H). ^13^C NMR (100 MHz, CDCl_3_): δ 165.80, 149.34, 145.73, 144.48, 142.46, 142.01, 138.19, 132.85, 129.86, 129.49, 128.68, 128.18, 128.17, 127.99, 126.68, 126.17, 116.83, 72.96, 41.50, 36.51, 36.23, 35.37, 35.36, 34.27, 34.14, 34.04, 33.87, 32.07, 32.03, 29.21, 29.04, 27.82, 25.98, 22.76, 22.58, 22.48, 20.59, 20.04, 19.58, 18.91, 14.46, 14.34, 14.20, 11.57, 9.00. HR-MS (ESI^+^): *m/z* [M+ H]^+^ calculated for C_32_H_37_O_2_, 453.2788, found 453.2780.

**17 (bexarotene 1-ethylpropyl ester; pentan-3-yl 4-(1-(3,5,5,8,8-pentamethyl-5,6,7,8-tetrahydronaphthalen-2-yl)vinyl)benzoate).** General synthetic scheme for esterification of BEX was used with 3-pentanol as the reactant. The final compound was obtained as colourless oil (79 mg, 65.8% yield). Purity was >98% by HPLC-UV. ^1^H NMR (400 MHz, CDCl_3_): δ 8.00 (dt, *J_1_* = 8.5 Hz, *J_2_* =1.8 Hz, 2H), 7.37 (dt, *J_1_* = 8.5 Hz, *J_2_* = 1.8 Hz, 2H), 7.15 (s, 1H), 7.10 (s, 1H), 5.83 (d, *J* = 1.3 Hz, 1H), 5.34 (d, *J* = 1.3 Hz, 1H), 5.03 (quintet, *J* = 6.1 Hz, 1H), 1.98 (s, 3H), 1.73 (s, 4H), 1.72 (quintet, *J* = 7.4 Hz, 4H), 1.33 (s, 6H), 1.30 (s, 6H), 0.97 (t, *J* = 7.4 Hz, 6H). ^13^C NMR (100 MHz, CDCl_3_): δ 166.46, 149.38, 145.48, 144.45, 142.44, 138.25, 132.88, 129.85, 129.73, 128.18, 128.15, 126.63, 116.70, 41.51, 36.52, 36.24, 35.39, 35.37, 34.28, 34.14, 34.04, 33.88, 32.08, 32.04, 29.22, 29.05, 27.83, 26.70, 25.98, 22.76, 22.49, 20.59, 20.06, 19.58, 18.91, 14.46, 14.20, 11.57, 9.77, 9.00. HR-MS (ESI^+^): *m/z* [M+ H]^+^ calculated for C_29_H_39_O_2_, 419.2945, found 419.2936.

**18 (bexarotene 1-isopropyl-2-methyl-propyl ester; 2,4-dimethylpentan-3-yl 4-(1-(3,5,5,8,8-pentamethyl-5,6,7,8-tetrahydronaphthalen-2-yl)vinyl)benzoate).** General synthetic scheme for esterification of BEX was used with 2,4-dimethyl-3-pentanol as the reactant. The final compound was obtained as colourless oil (117 mg, 91.3% yield). Purity was >98% by HPLC-UV. ^1^H NMR (400 MHz, CDCl_3_): δ 8.00 (dt, *J_1_* = 8.5 Hz, *J_2_* =1.8 Hz, 2H), 7.37 (dt, *J_1_* = 8.4 Hz, *J_2_* = 1.8 Hz, 2H), 7.14 (s, 1H), 7.10 (s, 1H), 5.83 (d, *J* = 1.2 Hz, 1H), 5.34 (d, *J* = 1.2 Hz, 1H), 4.85 (t, *J* = 6.1 Hz, 1H), 2.10-1.95 (m, 5H), 1.72 (s, 4H), 1.32 (s, 6H), 1.30 (s, 6H), 0.96 (d, *J* = 6.7 Hz, 12H). ^13^C NMR (100 MHz, CDCl_3_): δ 166.63, 149.36, 145.46, 144.45, 142.45, 138.25, 132.89, 129.83, 129.67, 128.18, 128.15, 126.65, 116.71, 83.33, 41.51, 36.52, 36.24, 35.37, 34.28, 34.15, 34.05, 33.88, 32.09, 32.04, 29.83, 29.22, 29.05, 27.83, 25.99, 22.77, 22.49, 20.59, 20.07, 19.79, 19.59, 18.92, 17.51, 14.47, 14.21, 11.58, 9.00. HR-MS (ESI^+^): *m/z* [M+ H]^+^ calculated for C_31_H_43_O_2_, 447.3258, found 447.3255.

**19 (bexarotene N,N-dimethylacetamide ester; 2-(dimethylamino)-2-oxoethyl 4-(1-(3,5,5,8,8-pentamethyl-5,6,7,8-tetrahydronaphthalen-2-yl)vinyl)benzoate).** BGA1 was synthesised by adding 0.344 mmol of 2-chloro-N,N-dimethylacetamide to 0.287 mmol of BEX. Catalysts of 0.412 mmol KI and 0.344 mmol K_2_CO_3_ were added and 4 mL of DMF was used as the solvent. The mixture was stirred at 100 °C using a magnetic stirrer overnight. Purification was performed by flash chromatography using hexane:ethyl acetate = 7:3 (v/v) as the mobile phase. The final compound was obtained as white solid (42.5% yield), melting point 134-136 °C. Purity was >97% by HPLC-UV. ^1^H NMR (400 MHz, CDCl_3_): δ 8.05 (dt, *J_1_* = 8.4 Hz, *J_2_* =1.8 Hz, 2H), 7.36 (dt, *J_1_* = 8.4 Hz, *J_2_* = 1.8 Hz, 2H), 7.14 (s, 1H), 7.09 (s, 1H), 5.82 (d, *J* = 1.2 Hz, 1H), 5.34 (d, *J* = 1.2 Hz, 1H), 4.96 (s, 2H), 3.06 (s, 3H), 3.01 (s, 3H), 1.95 (s, 3H), 1.71 (s, 4H), 1.32 (s, 6H), 1.29 (s, 6H). ^13^C NMR (100 MHz, CDCl_3_): δ 171.30, 166.60, 166.26, 149.35, 146.11, 144.49, 142.45, 138.14, 132.90, 130.19, 128.43, 128.22, 128.18, 126.75, 117.03, 61.98, 60.54, 41.50, 36.11, 35.76, 35.36, 34.14, 34.03, 29.20, 27.81, 13.97, 22.75, 21.17, 20.58, 20.07, 19.57, 18.90, 14.45, 14.33, 11.56. HR-MS (ESI^+^): *m/z* [M+ H]^+^ calculated for C_28_H_36_NO_3_, 434.2690, found 434.2687.

**20 (bexarotene N,N-diethylacetamide ester;** **2-(diethylamino)-2-oxoethyl 4-(1-(3,5,5,8,8-pentamethyl-5,6,7,8-tetrahydronaphthalen-2-yl)vinyl)benzoate).** BGA2 was synthesised by adding 0.344 mmol of 2-chloro-N,N-diethylacetamide to 0.287 mmol of BEX. Catalysts of 0.412 mmol KI and 0.344 mmol K_2_CO_3_ were added and 4 mL of DMF was used as the solvent. The mixture was stirred at 100 °C using a magnetic stirrer overnight. Purification was performed by flash chromatography using DCM:MeOH with 0.7 N NH_3_ = 50:2 (v/v) as the mobile phase. The final compound was obtained as white solid (59 mg, 29.7% yield), melting point 146-148 °C. Purity was >98% by HPLC-UV. ^1^H NMR (400 MHz, CDCl_3_): δ 8.05 (dt, *J_1_* = 8.5 Hz, *J_2_* =1.8 Hz, 2H), 7.36 (dt, *J_1_* = 8.5 Hz, *J_2_* =1.8 Hz, 2H), 7.14 (s, 1H), 7.09 (s, 1H), 5.82 (d, *J* = 1.2 Hz, 1H), 5.34 (d, *J* = 1.2 Hz, 1H), 4.96 (s, 2H), 3.44 (q, *J* = 7.2 Hz, 2H), 3.33 (q, *J* = 7.2 Hz, 2H), 1.95 (s, 3H), 1.71 (s, 4H), 1.32 (s, 6H), 1.29 (s, 6H), 1.27 (t, *J* = 7.2 Hz, 3H), 1.17 (t, *J* = 7.2 Hz, 3H). ^13^C NMR (100 MHz, CDCl_3_): δ 166.29, 165.70, 149.34, 146.04, 144.46, 142.42, 138.13, 132.88, 130.16, 128.49, 128.20, 128.16, 126.72, 117.00, 61.94, 53.55, 41.19, 40.61, 35.35, 35.33, 34.11, 34.01, 32.04, 32.00, 23.95, 20.04, 14.35, 13.04. HR-MS (ESI^+^): *m/z* [M+ H]^+^ calculated for C_30_H_40_NO_3_, 462.3003, found 462.3007.

**21 (bexarotene isopropyl carbonate ethyl ester; 1-((isopropoxycarbonyl)oxy)ethyl 4-(1-(3,5,5,8,8-pentamethyl-5,6,7,8-tetrahydronaphthalen-2-yl)vinyl)benzoate).** BAM1 was synthesised by adding 0.344 mmol of 1-chloroethyl isopropyl carbonate to 0.287 mmol of BEX. Catalysts of 0.412 mmol KI and 0.344 mmol K_2_CO_3_ were added and 4 mL of DMF was used as the solvent. The mixture was stirred at 65 °C using a magnetic stirrer for 2 h. Purification was performed by the general synthetic scheme. The final compound was obtained as colourless oil (45.6% yield). Purity was >97% by HPLC-UV. ^1^H NMR (400 MHz, CDCl_3_): δ 7.99 (dt, *J_1_* = 8.5 Hz, *J_2_* =1.7 Hz, 2H), 7.36 (dt, *J_1_* = 8.5 Hz, *J_2_* =1.7 Hz, 2H), 7.14 (s, 1H), 7.10 (s, 1H), 7.04 (q, *J* = 5.4 Hz, 1H), 5.83 (d, *J* = 1.2 Hz, 1H), 5.35 (d, *J* = 1.2 Hz, 1H), 4.93 (septet, *J* = 6.2 Hz, 1H), 1.96 (s, 3H), 1.72 (s, 4H), 1.66 (d, 5.4 Hz, 3H), 1.33 (d, 6.2 Hz, 6H), 1.32 (s, 6H), 1.30 (s, 6H). ^13^C NMR (100 MHz, CDCl_3_): δ 164.51, 152.67, 149.26, 146.32, 144.55, 142.49, 138.06, 132.83, 130.15, 128.20, 128.16, 126.76, 117.18, 91.76, 72.84, 53.55, 35.36, 35.34, 34.14, 34.04, 32.07, 32.02, 21.82, 21.78, 20.05, 19.88. HR-MS (ESI^+^): *m/z* [M+ Na]^+^ calculated for C_30_H_38_NaO_5_, 501.2611, found 501.2611.

**22 (bexarotene cyclohexyl carbonate ethyl ester; 1-(((cyclohexyloxy)carbonyl)oxy)ethyl 4-(1-(3,5,5,8,8-pentamethyl-5,6,7,8-tetrahydronaphthalen-2-yl)vinyl)benzoate).** BAM2 was synthesised by adding 0.344 mmol of 1-chloroethyl cyclohexyl carbonate to 0.287 mmol of BEX. Catalysts of 0.412 mmol KI and 0.344 mmol K_2_CO_3_ were added and 4 mL of DMF was used as the solvent. The mixture was stirred at 65 °C using a magnetic stirrer for 2 h. Purification was performed by the general synthetic scheme. The final compound was obtained as colourless oil (109 mg, 48.8% yield). Purity was >98% by HPLC-UV. ^1^H NMR (400 MHz, CDCl_3_): δ 7.99 (dt, *J_1_* = 8.5 Hz, *J_2_* =1.7 Hz, 2H), 7.36 (dt, *J_1_* = 8.5 Hz, *J_2_* =1.7 Hz, 2H), 7.14 (s, 1H), 7.10 (s, 1H), 7.04 (q, *J* = 5.4 Hz, 1H), 5.83 (d, *J* = 1.2 Hz, 1H), 5.35 (d, *J* = 1.2 Hz, 1H), 4.66 (septet, *J* = 4.7 Hz, 1H), 1.99-1.90 (m, 2H), 1.95 (s, 3H), 1.82-1.69 (m, 2H), 1.72 (s, 4H), 1.66 (d, 5.4 Hz, 3H), 1.65-1.43 (m, 6H), 1.32 (s, 6H), 1.29 (s, 6H). ^13^C NMR (100 MHz, CDCl_3_): δ 164.51, 152.67, 149.26, 146.30, 144.54, 142.49, 138.07, 132.83, 130.15, 128.19, 126.76, 117.16, 91.79, 60.53, 41.50, 35.36, 35.34, 34.14, 34.03, 32.06, 32.02, 31.55, 31.53, 29.20, 25.30, 23.73, 22.75, 20.05, 19.88, 14.45, 14.34, 11.56. HR-MS (ESI^+^): *m/z* [M+ Na]^+^ calculated for C_33_H_42_NaO_5_, 541.2924, found 541.2939.

**23 (bexarotene dimethylaminoethyl ester; 2-(dimethylamino)ethyl 4-(1-(3,5,5,8,8-pentamethyl-5,6,7,8-tetrahydronaphthalen-2-yl)vinyl)benzoate).** General synthetic scheme for esterification of BEX was used with 2-dimethylaminoethanol as the reactant. Flash chromatography was performed with DCM:MeOH with 0.7 N NH_3_ = 50:2 as the mobile phase for purification of the product. The final compound was obtained as yellowish gum (73.1% yield). Purity was >97% by HPLC-UV. ^1^H NMR (400 MHz, CDCl_3_): δ 7.98 (dt, *J_1_* = 8.5 Hz, *J_2_* =1.8 Hz, 2H), 7.36 (dt, *J_1_* = 8.5 Hz, *J_2_* =1.8 Hz, 2H), 7.14 (s, 1H), 7.09 (s, 1H), 5.82 (d, *J* = 1.3 Hz, 1H), 5.34 (d, *J* = 1.2 Hz, 1H), 4.46 (t, *J* = 5.8 Hz, 2H), 2.76 (t, *J* = 5.8 Hz, 2H), 2.38 (s, 6H), 1.95 (s, 3H), 1.72 (s, 4H), 1.32 (s, 6H), 1.29 (s, 6H). ^13^C NMR (100 MHz, CDCl_3_): δ 166.54, 149.32, 145.79, 144.48, 142.45, 138.15, 132.84, 129.85, 129.07, 128.18, 128.16, 126.70, 116.91, 62.92, 57.89, 53.54, 45.86, 35.35, 35.34, 34.13, 34.02, 32.06, 32.01, 20.04. HR-MS (ESI^+^): *m/z* [M+ H]^+^ calculated for C_28_H_38_NO_2_, 420.2897, found 420.2909.

**24 (bexarotene diisopropylaminoethyl ester; 2-(diisopropylamino)ethyl 4-(1-(3,5,5,8,8-pentamethyl-5,6,7,8-tetrahydronaphthalen-2-yl)vinyl)benzoate).** General synthetic scheme for esterification of BEX was used with 2-diisopropylaminoethanol as the reactant. Flash chromatography was performed with DCM:MeOH with 0.7 N NH_3_ = 50:2 as the mobile phase for purification of the product. The final compound was obtained as yellowish gum (80 mg, 36.5% yield). Purity was >97% by HPLC-UV. ^1^H NMR (400 MHz, CDCl_3_): δ 7.98 (dt, *J_1_* = 8.5 Hz, *J_2_* =1.8 Hz, 2H), 7.36 (dt, *J_1_* = 8.5 Hz, *J_2_* =1.8 Hz, 2H), 7.14 (s, 1H), 7.09 (s, 1H), 5.82 (d, *J* = 1.2 Hz, 1H), 5.34 (d, *J* = 1.2 Hz, 1H), 4.27 (broad s, 2H), 3.07 (broad s, 2H), 2.81 (broad s, 2H), 1.96 (s, 3H), 1.72 (s, 4H), 1.32 (s, 6H), 1.29 (s, 6H), 1.07 (broad s, 12H). ^13^C NMR (100 MHz, CDCl_3_): δ 166.63, 149.36, 144.48, 142.45, 138.19, 132.87, 129.79, 128.21, 128.17, 126.69, 116.88, 53.55, 49.50, 43.95, 35.36, 35.35, 34.14, 34.03, 32.07, 32.03, 20.99, 20.07. HR-MS (ESI^+^): *m/z* [M+ H]^+^ calculated for C_32_H_46_NO_2_, 476.3523, found 476.3536.

**25 (bexarotene (5-methyl-2-oxo-1,3-dioxolen-4-yl)methyl ester; (5-methyl-2-oxo-1,3-dioxol-4-yl)methyl 4-(1-(3,5,5,8,8-pentamethyl-5,6,7,8-tetrahydronaphthalen-2-yl)vinyl)benzoate).** General synthetic scheme for esterification of BEX was used with (5-methyl-2-oxo-1,3-dioxolen-4-yl)methyl alcohol as the reactant. Purification was performed by using hexane:EA = 88:12 as the mobile phase. The final compound was obtained as white solid (170 mg, 85.6% yield), melting point 96-98 °C. Purity was >98% by HPLC-UV. ^1^H NMR (400 MHz, CDCl_3_): δ 7.98 (dt, *J_1_* = 8.5 Hz, *J_2_* =1.7 Hz, 2H), 7.38 (dt, *J_1_* = 8.5 Hz, *J_2_* =1.7 Hz, 2H), 7.14 (s, 1H), 7.10 (s, 1H), 5.84 (d, *J* = 1.2 Hz, 1H), 5.36 (d, *J* = 1.2 Hz, 1H), 5.09 (s, 2H), 2.26 (s, 3H), 1.96 (s, 3H), 1.72 (s, 4H), 1.32 (s, 6H), 1.29 (s, 6H). ^13^C NMR (100 MHz, CDCl_3_): δ 171.28, 165.97, 152.27, 149.17, 146.47, 144.58, 142.51, 140.30, 137.98, 133.80, 132.80, 130.04, 128.21, 128.18, 127.92, 126.87, 117.31, 60.52, 54.24, 35.33, 35.31, 34.13, 34.02, 32.05, 32.00, 23.96, 21.16, 20.04, 14.32, 9.57. HR-MS (ESI^+^): *m/z* [M+ H]^+^ calculated for C_29_H_33_O_5_, 461.2323, found 461.2323.

**26 (retinoic acid ethyl ester; ethyl (2E,4E,6E,8E)-3,7-dimethyl-9-(2,6,6-trimethylcyclohex-1-en-1-yl)nona-2,4,6,8-tetraenoate).** General synthetic scheme for esterification of RA was used with ethanol as the reactant. Purification was performed by using [(hexane:toluene, 50:50)]:ethyl acetate = 50:2 as the mobile phase. The final compound was obtained as yellow gum (70.6% yield). Purity was >98% by HPLC-UV. ^1^H NMR (400 MHz, CDCl_3_): δ 7.09 – 6.96 (m, 1H), 6.35 – 6.24 (m, 2H), 6.21 – 6.12 (m, 2H), 5.82 – 5.77 (m, 1H), 4.20 (q, *J* = 7.1 Hz, 2H), 2.12 – 1.97 (m, 3H), 2.06 – 1.99 (m, 3H), 1.76 – 1.70 (m, 3H), 1.71 – 1.59 (m, 2H), 1.55 – 1.43 (m, 2H), 1.32 (t, *J* = 7.1 Hz, 3H), 1.05 (s, 6H). ^13^C NMR (100 MHz, CDCl_3_): δ 167.21, 152.70, 139.54, 137.70, 137.48, 137.28, 135.18, 132.13, 130.91, 130.35, 129.99, 129.50, 129.34, 128.63, 128.46, 118.60, 116.59, 59.64, 39.61, 34.27, 33.11, 28.96, 21.74, 19.23, 14.36, 13.83, 12.90. HR-MS (ESI^+^): *m/z* [M+ H]^+^ calculated for C_22_H_33_O_2_, 329.2475, found 329.2476.

**27 (retinoic acid (5-methyl-2-oxo-1,3-dioxolen-4-yl)methyl ester; (5-methyl-2-oxo-1,3-dioxol-4-yl)methyl (2E,4E,6E,8E)-3,7-dimethyl-9-(2,6,6-trimethylcyclohex-1-en-1-yl)nona-2,4,6,8-tetraenoate).** General synthetic scheme for esterification of RA was used with (5-methyl-2-oxo-1,3-dioxolen-4-yl)methyl alcohol as the reactant. Purification was performed by using hexane:EA = 90:10 as the mobile phase. The final compound was obtained as yellow gum (68.5% yield). Purity was >98% by HPLC-UV. ^1^H NMR (400 MHz, CDCl_3_): δ 7.07 (dd, *J*_1_ = 11.4 Hz, *J*_2_ = 15.0 Hz, 1H), 6.37 – 6.25 (m, 2H), 6.24 – 6.12 (m, 2H), 5.79 (s, 1H). ^13^C NMR (100 MHz, CDCl_3_): δ 207.07, 166.19, 154.99, 152.26, 140.41, 139.71, 137.65, 137.15, 134.58, 133.97, 133.27, 132.00, 130.23, 130.11, 129.31, 129.15, 129.03, 117.48, 116.63, 114.61, 52.97, 39.60, 39.53, 34.27, 33.12, 33.06, 30.93, 28.95, 21.74, 21.08, 19.20, 14.05, 12.95, 9.40. HR-MS (ESI^+^): *m/z* [M+ H]^+^ calculated for C_25_H_33_O_5_, 413.2323, found 413.2308.

**
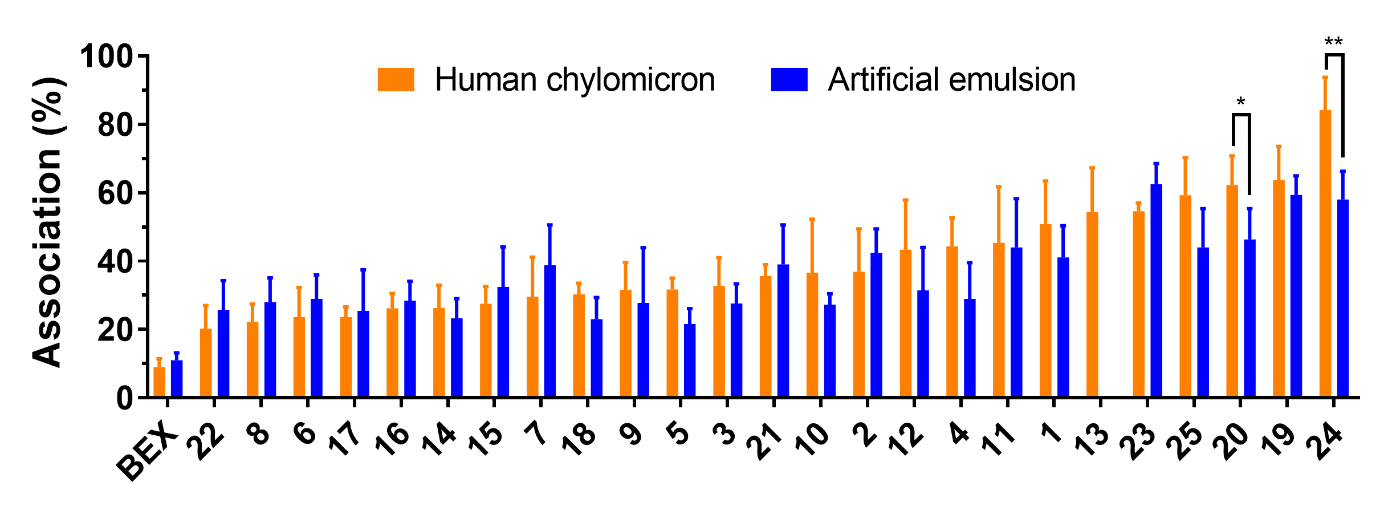
**

**Supplementary Material Fig. S1. Association of bexarotene (BEX) and its prodrugs with artificial emulsion prepared from Intralipid^®^ and natural human chylomicrons (CM) representing potential of intestinal lymphatic system targetability (mean ± SD, n = 5).** *, p<0.05; **, p<0.01.

**
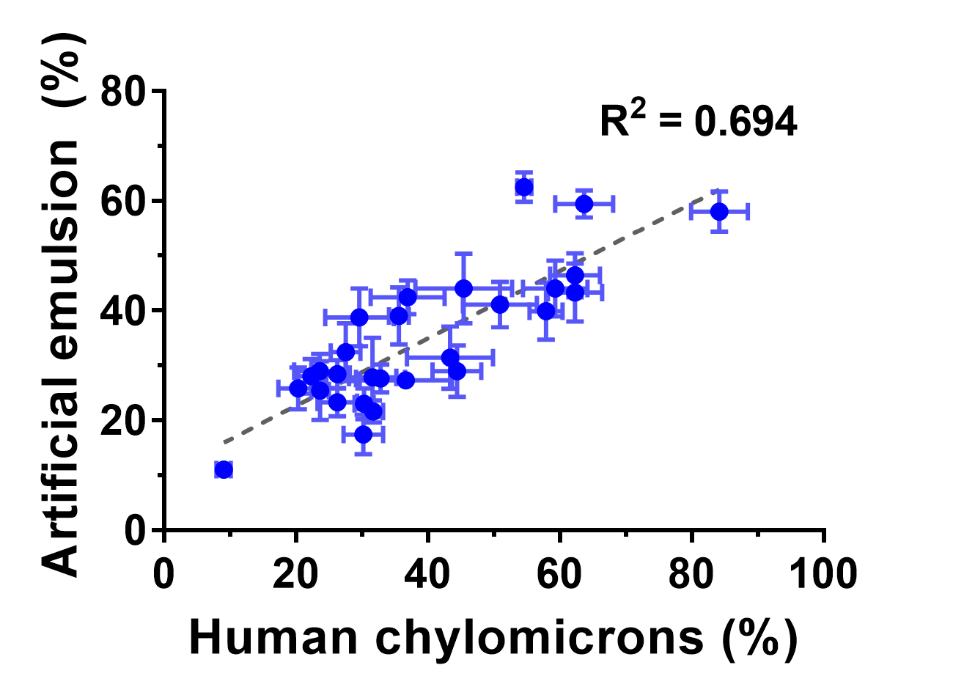
**

**Supplementary Material Fig. S2.** **Correlation graph between association of compounds with human chylomicrons and artificial emulsions prepared from Intralipid^®^ (mean ± SD).** **Moderate correlation with coefficient of correlation (*R^2^*) of 0.694 was found between the two test media.**

**Supplementary Material Fig. S3.** **Degradation half-lives of prodrugs of bexarotene (BEX) in rat plasma and fasted state simulated intestinal fluid (FaSSIF) with added esterase activity (20 IU/mL), representing their converting rate to BEX (mean ± SD, n = 3).**

**Supplementary Material 2. *In vitro* efficacy results of BEX and RA**

The anticancer effects of BEX and RA were assessed by MTT assay using DLBCL cell lines. The *GI*_50_ of each drug was firstly determined in each cell line. Subsequently, cells were co-treated with different doses of RA and a range of BEX concentrations in order to obtain the *GI*_50_ of BEX in the presence of RA. Cell growth was significantly and synergistically inhibited by BEX in conjunction with RA at low doses in both cell lines (0.625% × *GI_50_* of RA: 0.425 and 0.400 μM for DOHH2 and VAL, respectively), which is shown by the shift in dose-response curves (Supplementary Material Fig. S4a). The effective synergy in cell growth inhibition between the two drugs is shown as isobole plots following a previously reported method (Supplementary Material Fig. S4b). Synergy is demonstrated by the *GI*_50_ values of BEX in the presence of RA (orange dots), all of which are significantly lower than the ‘expected’ concentrations equivalent to an additive effect (blue line). Significantly reduced *GI*_50_ values of BEX when RA was co-administered also demonstrates improved potency and synergy of BEX and RA in DLBCL (Supplementary Material Fig. S4c).

**
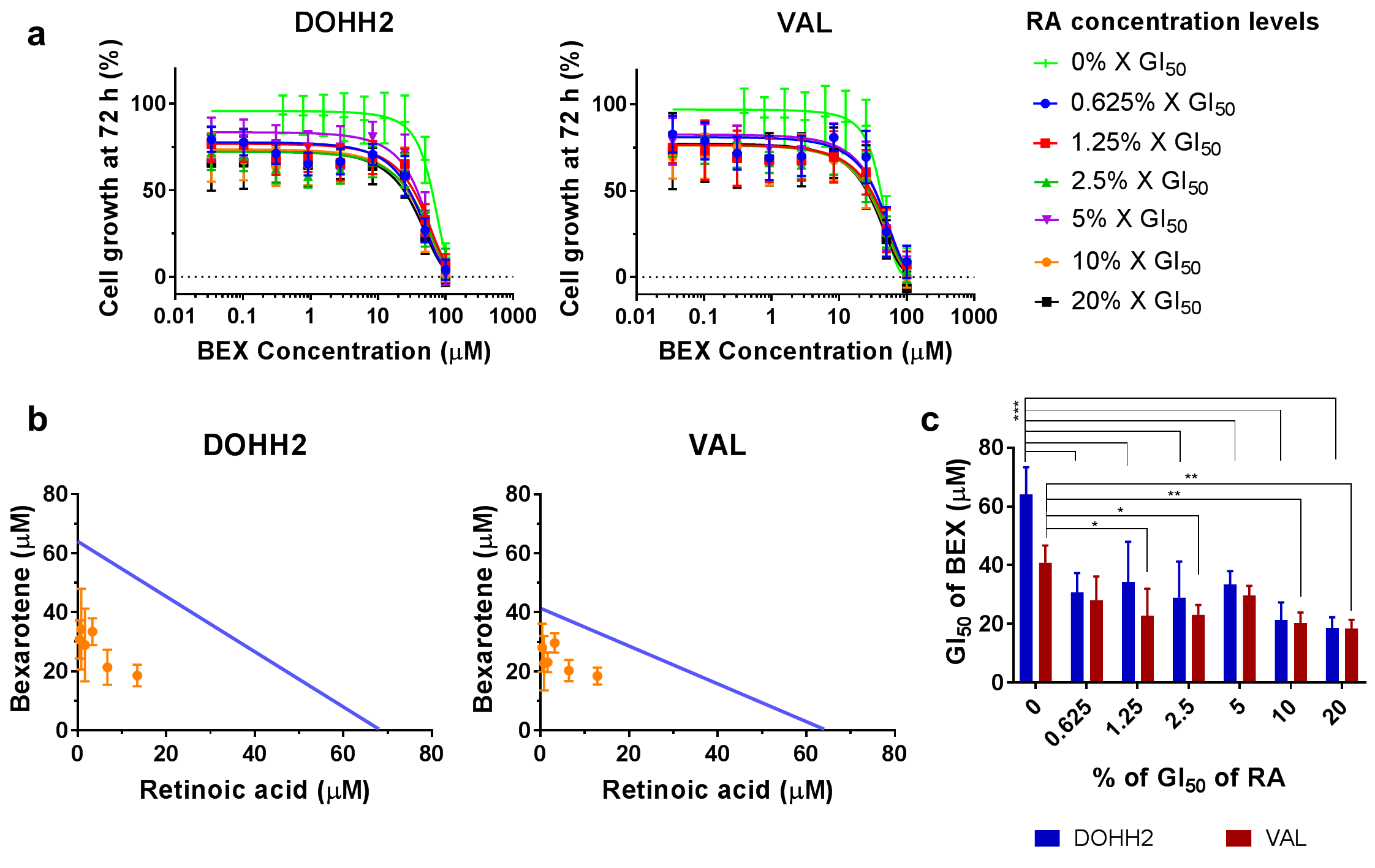
**

**Supplementary Material Fig. S4. Assessment of cell growth inhibition and synergism of bexarotene (BEX) and retinoic acid (RA) using MTT assay in the diffuse large B-cell lymphoma (DLBCL) cell lines DOHH2 and VAL. (a)** Dose-response curves showing cell growth inhibition of BEX in the presence of various concentrations of RA. **(b)** Isobolograms indicating effective synergism between BEX and RA. Blue lines represent expected effectiveness when the two drugs are additive, where the *y*-intercept is *GI*_50_ of BEX and *x*-intercept is *GI_5_*_0_ of RA. Orange dots represent the *GI*_50_ values observed with synergism (mean ± SD). **(c)** Synergism expressed by reduced *GI*_50_ of BEX in the presence of RA. All MTT assay results shown from 4 trials of n = 6 (mean ± SD).*, p<0.05; **, p<0.01; ***, p<0.001.

**Supplementary Material Table S1.** TG measurements in each test medium used for further conversion studies of **2** and **25** (mg/mL, n = 3)

|  |  | **AVG** | **SD** |
| --- | --- | --- | --- |
| **Blank rat plasma** | | 0.31 | 0.01 |
| **Rat plasma spiked with Intralipid^®^** | | 0.33 | 0.02 |
| **Postprandial rat lymph** | | 4.05 | 1.58 |

**Supplementary Material Table S2. Analytical conditions (HPLC-UV) for each compound**

| ***Compounds*** | **Moblie phase (%)** | | **Column^b^** | **Flow rate (mL/min)** | **pH modifier** | **Internal standard** | **Reconstitution solvent^c^** |
| --- | --- | --- | --- | --- | --- | --- | --- |
|  | **Buffer^a^** | **Acetonitrile** |  |  |  |  |  |
| BEX, **1**-**5**, **8**, **9**, **14**-**18**, **21**, **22** | 15 | 85 | C18 | 0.4 | 0.1 M HCl | DDT^d^ | 90% |
| RA, **26**, **27** | 15 | 85 | C18 | 0.4 | 0.1 M HCl | Probucol | 50% |
| **7** | 40 | 60 | C18 | 0.4 | 0.1 M HCl | DDT | 50% |
| **23** | 40 | 60 | C18 | 0.4 | None | DDT | 50% |
| **24** | 20 | 80 | C18 | 0.4 | None | DDT | 80% |
| **19** | 40 | 60 | C18 | 0.4 | 0.1 M HCl | DDT | 50% |
| **20**, **25** | 20 | 80 | C18 | 0.4 | 0.1 M HCl | DDT | 50% |
| **10**-**12** | Gradient 1^e^ | | Biphenyl | 0.6 | 0.1 M HCl | Probucol | 100% |
| **13** | Gradient 2^f^ | | Biphenyl | 0.6 | 0.1 M HCl | Probucol | 100% |

^a^ Buffer: 10 mM ammonium acetate, pH 4.1 (modified with glacial acetic acid)

^b^ C18: Gemini C18 2.0 × 150 mm, 3 µm particle size; Biphenyl: Kinetex Biphenyl 2.1 × 100 mm, 5 µm particle size.

^c^ Reconstitution solvent expressed as % of acetonitrile in water

^d^ DDT: dichlorodiphenyltrichloroethane

^e^ Gradient 1: Buffer 40% at 0-4 min, decrease to 5% at 4-7 min, 5% at 7-23 min, increase to 40% at 23-26 min, 40% at 26-45 min.

^f^ Gradient 2: Buffer 40% at 0-4 min, decrease to 5% at 4-7 min, decrease to 0% at 7-10 min, 0% at 10-25 min, increase to 40% at 25-27 min, 40% at 27-50 min.
